# A feasibility study to assess the recruitment and retention of pregnant patients who regularly use cannabis

**DOI:** 10.1186/s13104-024-06826-4

**Published:** 2024-06-25

**Authors:** Alyssa Vanderziel, Mark M. Maslovich, Omayma Alshaarawy

**Affiliations:** 1 Department of Family Medicine, College of Human Medicine, Michigan State University, East Lansing, MI USA; 2 Department of Epidemiology & Biostatistics, College of Human Medicine, Michigan State University, East Lansing, MI USA; 3 Center for Health Policy & Health Services Research, Henry Ford Health, Detroit, MI USA

**Keywords:** Pregnancy, Cannabis, Prenatal cannabis use, Recreational legalization, Michigan, Feasibility study, Prospective study

## Abstract

**Objective:**

To assess first-trimester recruitment and retention of pregnant patients who regularly used cannabis, but not other substances, measured by willingness to participate in a research study, completion of self-administered electronic questionnaires, and willingness to provide urine samples during each trimester of pregnancy. We designed and launched a prospective feasibility study titled, *Ca*nnabis *L*egalization in *M*ichigan (CALM) – *M*aternal & *I*nfant *H*ealth (MIH), in two Michigan clinics after the recreational use of cannabis became legal for adults 21 years and older.

**Results:**

Over half (52%) of patients asked to participate in CALM-MIH were consented to the study. Two-thirds (66%) of screened patients initiated prenatal care during their first trimester of pregnancy and 50% used cannabis, of which the majority did not concurrently use other substances. Of those recruited into the prospective study, all participants completed the first-trimester questionnaire and provided urine samples. Study retention was 80% and all participants who completed follow-up assessments were willing to provide urine samples.

**Supplementary Information:**

The online version contains supplementary material available at 10.1186/s13104-024-06826-4.

## Introduction

The prevalence of prenatal cannabis use has nearly doubled in the United States (**US**), from < 3% in 2002 to ∼ 5% in 2019 [1, 2], and the potency of cultivated cannabis has increased in the last decade [3]. Our analysis of data from the National Survey on Drug Use and Health (**NSDUH**) revealed that 3 in 4 pregnant people in the US do not characterize regular cannabis use as a great health risk [1]. Therefore, rigorous research on the motives of use, consequences of prenatal cannabis exposure, and variability in THC concentration is imperative to inform public health policies and guide clinical practice.

While some studies conclude null findings [4–6], others suggest statistically significant links between prenatal cannabis exposure and neonatal outcomes [7–9]. Limitations of prior research include cross-sectional designs and retrospective data collection, which may incite recall bias. In some studies, data were obtained from electronic health records and limited by the information captured. Further, previous research often lacked specificity of cannabis use metrics such as frequency and mode of use and trimester-specific timing, which allow for granular assessment of cannabis use on fetal development. Many studies are also limited by the effects of confounding from polysubstance use, particularly tobacco.

High quality, prospective studies are essential to assess the health effects of prenatal cannabis use. We designed a prospective feasibility study titled, *CannabisLegalization in Michigan (****CALM****) – Maternal & Infant Health (****MIH****).* The objectives were (1) to assess the recruitment of pregnant patients measured by willingness to participate in a study about prenatal cannabis use (Part A) and (2) the retention of first-trimester pregnant patients who regularly used cannabis, but not other substances, measured by willingness to participate, completion of self-administered electronic questionnaires at four timepoints throughout pregnancy and postpartum, and willingness to provide urine samples at each trimester of pregnancy (Part B).

## Methods

### Study design

This observational prospective study consisted of a non-probability convenience sample of pregnant patients and included an assessment in the first, second, and third trimesters, and within one month postpartum. The study began in October 2020 in Women’s Health and Family Medicine Residency clinics at Sparrow Health, Lansing, Michigan. These clinics care for > 650 pregnant patients/year, with a payer mix that is approximately 70% public and 30% private. Over 50% of the patients are African American. Recruitment continued through August 2021, with several pauses owing to the statewide COVID-19 Executive Order.

At each clinic, healthcare professionals were educated on study goals and eligibility criteria. CALM-MIH staff were provided with patient schedules for each week, excluding all personal identifiers. Additionally, the study was advertised via flyers at both clinics; patients interested in participation could contact CALM-MIH staff directly.

### Recruitment

To be eligible for screening, patients must have been pregnant, 21–35 years old, and initiating prenatal care. The restricted age group was in consideration of the legal age to use cannabis in Michigan (21 years) and to minimize the confounding effects of advanced maternal age on pregnancy outcomes. We restricted eligibility to patients initiating prenatal care to capture patients ≤ 3 months pregnant.

**Fig. 1:**
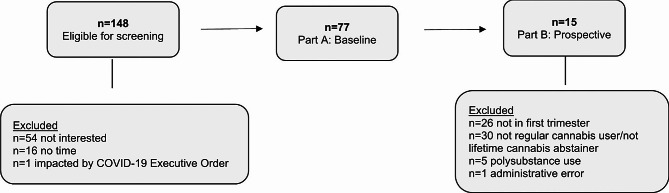
CALM-MIH Eligibility Flowchart

Screening and recruitment took place during the prenatal care appointment. Healthcare staff advertised the study to eligible patients (Fig. 1). If a patient expressed interest, they were directed to CALM-MIH staff to review informed consent. The patient selected an unmarked envelope containing a copy of the informed consent, a study business card, and a label sheet with a randomly generated unique identification (**ID**) number. To maintain privacy, written consent was not obtained; drawing a random envelope and agreeing to self-administer the study survey represented the consent mechanism.

The participant used an electronic device to complete a self-administered Qualtrics questionnaire (Supplemental Survey 1). The first survey item required the participant to enter their unique ID, followed by questions about general health, previous pregnancies, substance use, and demographic information. No personal identifying information was collected. The purpose of this survey was (1) to collect descriptive baseline data on all participants (Part A) and (2) to determine eligibility for prospective follow-up (Part B). Participants who were ineligible for prospective follow-up received a $10 gift card to thank them for their time.

### Prospective follow-up

Eligibility for follow-up included **a**) first-trimester pregnancy, **b**) either no history of cannabis use or regular cannabis use (i.e., used cannabis ≥ 4 days in the 30 days prior to assessment), **c**) did not use any tobacco products in the past 30 days, **(d)** drank no more than one alcoholic beverage on the days they had alcohol in the past 30 days, and **(e)** did not use cocaine, heroin, methadone, methamphetamine, benzodiazepines, or prescription pain relivers (i.e., in a way not directed by the prescribing provider) in the past 30 days.

Participants who had never used cannabis were recruited to prevent identification of cannabis users by study and healthcare staff. Participants were asked to provide a urine sample, to assess concordance between self-reported substance use and toxicology results, and to complete an additional questionnaire to schedule their second-trimester assessment. An administrative assistant, who did not have access to research data, maintained all communication with participants. The second-trimester assessment (Supplemental Survey 2) was conducted during the participant’s obstetric appointment to enhance convenience and minimize time and effort commitment to the study activities. Similar procedures were followed for the third-trimester assessment. First-, second-, and third-trimester surveys included questions on general health, substance use, reasons for cannabis use, risk perception of using cannabis, and morning sickness.

The postpartum questionnaire (Supplemental Survey 3), which focused on pregnancy outcomes, was administered via e-mail using a link to the questionnaire and was completed within one month of delivery. During the third-trimester assessment, study staff supplied participants with an information sheet and explained the postpartum assessment, which asked for birthweight, gestational age, and Apgar scores. Staff advised participants to place the information sheet in their overnight bag for their hospital stay during delivery so that a nurse may assist in the completion of the requested information. The participants were thanked with a $25 gift card after each assessment, totaling up to $100 for participation.

### Urinalysis

After cannabis use, Δ9-tetrahydrocannabinol (**THC**) is metabolized to the inactive metabolite, 11-Nor-9-carboxy-Δ9-tetrahydrocannabinol (**THC-COOH**), and excreted in urine [10]. Urinary THC-COOH is very stable and cannabis use can be detected for several weeks [10]. First, we screened for THC-COOH in all urine samples using a competitive immunochemical assay with a cutoff of 50 ng/mL (Alere iCup® Dx). Next, we quantified THC-COOH and creatinine (to control for urine dilution) in the positively screened samples by Liquid chromatography/tandem mass spectrometry (**LC-MS/MS**).

We used the same immunochemical assay to screen for amphetamine, barbiturates, benzodiazepines, buprenorphine, oxycodone, cocaine, opiates, methamphetamine and methadone (Alere iCup® Dx). Additionally, we evaluated the presence of cotinine, a nicotine metabolite, in urine at the cut-off concentration of 200ng/mL (Abbott™ NicQuick™).

## Results

We identified 148 eligible patients, of which 64% (*n* = 94) expressed interest in the study. Among eligible patients, 52% (*n* = 77) consented to participate. Two-thirds of participants were in their first trimester (mean = 2.6 months) and the mean age was 26 years (SD ± 4). 91% (*n* = 70) reported a history of cannabis use, demonstrating the willingness of cannabis-using patients to participate in the study. Further, 49% had used cannabis in the 30 days prior to assessment. Table 1 illustrates additional descriptive characteristics.

We assessed the feasibility of prospective follow-up and collection of urine in a subsample of participants (*n* = 15). Four participants had no history of cannabis use, while 11 regularly used cannabis. Of regular users, 8 used cannabis ≥ 21 days in the 30 days prior to the assessment. Of first-, second-, and third-trimester participants who reported regular cannabis use (*n* = 11, *n* = 7, and *n* = 6, respectively), all used at least 1–2 times on the days they used cannabis. Study retention was 80%. Of the 15 first-trimester participants, 3 were lost-to-follow-up (1 cannabis user and 2 never users). Of the remaining 12 participants, 83% had complete data across all four timepoints, and all were willing to provide urine samples.

**Table 1:** Major characteristics of the study sample (*n* = 77)

**Maternal age, years**	Mean	Standard deviation
	26.4	4.0
	*N*	*%*
**Cannabis use**		
Recent user^a^	38	49.4
Past user^b^	32	41.5
Never user^c^	6	7.8
Missing	1	1.3
**Trimester**		
First	51	66.2
Second	19	24.7
Third	6	7.8
Missing	1	1.3
**Race/ethnicity**		
Non-Hispanic White	30	39.0
Non-Hispanic Black	27	35.0
Hispanic	13	16.9
Multiracial	7	9.1
**Education level**		
No high school diploma	11	14.3
High school diploma/GED/ some college	54	70.1
College or graduate degree	12	15.6
**Household income**		
<$25,000	47	61.0
$25,000-$49,999	22	28.6
$50,000-$74,999	5	6.5
≥$75,000	3	3.9
**Health insurance**		
Medicaid	59	76.6
Private	15	19.5
None/unsure	3	3.9
**Marital status**		
Married	21	27.3
Never married	39	50.6
Other	17	22.1
**Pre-pregnancy BMI**		
Obese	31	40.3
Not obese	46	59.7
**Gravidity** ^**†**^		
1–4 pregnancies	59	76.6
5 + pregnancies	18	23.4

^a^ Used cannabis in the past 30 days

^b^ Used cannabis in the past but not in the past 30 days

^c^ Never used cannabis in lifetime

^**†**^ Number of times pregnant including current pregnancy

Our results indicate 100% agreement between self-report and urine toxicology for all substances tested (Table 2). In the positively screened first-trimester urine samples, levels of THC-COOH ranged from 15 ng/mg creatinine (participant used cannabis on 5 days in the 30 days prior to assessment) to 2,414 ng/mg creatinine (participant used cannabis on all 30 days), and the mean THC-COOH concentration decreased from 607 ng/mg creatinine in the first trimester to 106 ng/mg creatinine in the third trimester.

**Table 2:** Concordance between self-report and urinalysis

	Self-reported Cannabis Use^a^			Self-reported Tobacco Use^b^			Self-reported Substance Use^c^	
	**Panel A: Trimester 1**							
**Urinalysis**	**Yes (Mean concentration)**	**No**		**Yes**	**No**		**Yes**	**No**
**Yes**	11 (607 ng/mg creatinine)	0		0	4		0	0
**No**	0	4		0	11		0	15
	**Panel B: Trimester 2**							
**Yes**	5 (369 ng/mg creatinine)	0		0	2		0	0
**No**	0	5		0	8		0	10
	**Panel C: Trimester 3**							
**Yes**	7 (106 ng/mg creatinine)	0		0	4		0	0
**No**	0	5		0	8		0	12

Agreement between self-reported tobacco use and urinary cotinine levels was lower (70%). This might be attributed to heavy second-hand smoke exposure, dishonest reporting, or the use of cannabis mixed with tobacco. Indeed, participants who screened positive for cotinine reported cannabis use predominantly as “smoked a cigar with marijuana in it, such as a blunt.”

## Discussion

Of eligible patients, the majority expressed interest in the CALM-MIH study and over half consented to participate. Nearly all screened participants had used cannabis at some point in their lifetime, which highlights the strong interest-level of pregnant patients who had a history of cannabis use to participate. However, several patients declined to participate, emphasizing that they did not want to take part in a ‘cannabis study.’ Thus, it is possible that stigma influences participation among nonusers.

The study retention rate (80%) was acceptable. Of those remaining in the sample, many participants had complete data across all timepoints, and all agreed to provide urine samples, confirming the feasibility of this study.

Due to small sample size, we were unable to assess exposure-outcome relationships. However, descriptive statistics revealed a high rate and frequency of prenatal cannabis use. The convenience sampling method used in this study may partially explain this high rate. Due to the legal status of cannabis in Michigan, an abundance of cannabis dispensaries are located in the Greater Lansing area. A study conducted by Dickenson and colleagues revealed that nearly 70% of Colorado dispensaries recommended first-trimester cannabis use to treat nausea [11]. It is possible that dispensaries in Michigan may be making similar recommendations.

It is worth noting that most patients who met eligibility screening criteria were in their first trimester, signifying a strength of our study in that it is possible to recruit patients early in pregnancy. Moreover, the 100% concordance rate between self-reported cannabis use and urine toxicology results reflect honest reporting.

## Limitations

Several lessons were learned from this study. Pregnant patients who verbally reported not using cannabis were often disinterested in participating, whereas those who reported cannabis use displayed eagerness to be involved in the study. Participants who reported having no history of cannabis use were different from those who regularly used cannabis in terms of socioeconomic factors. Future research might account for such factors at the design phase using matching or enrollment restriction. Further, a mixed method approach would have been beneficial such that qualitative interviews might have captured the full scope of responses by allowing participants to share their perspectives about using cannabis. Moreover, individuals who chose not to initiate prenatal care were not included in our sample and may differ from those who seek prenatal care in terms of cannabis use status.

The current study successfully addressed limitations of prior research by examining the feasibility of restricting the sample to first-trimester recruitment, excluding polysubstance use, and measuring regular cannabis use via self-report and urinalysis during each trimester of pregnancy. Future well-powered studies should restrict recruitment to the first trimester, as this is a critical period to capture the effects of substance use on fetal development. Additionally, future research might consider restricting the sample to pregnant participants who are only using cannabis, to parse out the confounding effects of other substance use. While urinalysis is the gold standard to detect substance use, our study indicates honest self-reporting in a state with legal recreational cannabis use, offering a less expensive and efficient option for substance use detection. Finally, this study might encourage future research to administer surveys with fine-grained assessment of cannabis use and include qualitative interviews to elicit a full range of perspectives and motives of using cannabis during pregnancy.

### Electronic supplementary material

Below is the link to the electronic supplementary material.

Supplementary Material 1

Supplementary Material 2

Supplementary Material 3

## Data Availability

To protect participant privacy, the descriptive data that support the findings of this study cannot be shared openly due to the small size of our sample. However, the data generated and analyzed may be made available by the corresponding author upon reasonable request.

## Abbreviations

US: United States
NSDUH: National Survey on Drug Use and Health
CALM-MIH: Cannabis Legalization in Michigan-Maternal & Infant Health
ID: Identification
THC: Δ9-tetrahydrocannabinol
THC-COOH: 11-Nor-9-carboxy-Δ9-tetrahydrocannabinol
LC-MS/MS: Liquid chromatography/tandem mass spectrometry
