# Supplementary material for: A feasibility study to assess the recruitment and retention of pregnant patients who regularly use cannabis
Source: BMC Res Notes. 2024 Jun 25;17:177. doi: 10.1186/s13104-024-06826-4 (PMC11197186; doi:10.1186/s13104-024-06826-4)
Supplement: Supplementary file 1 — Supplementary Material 1 [file 13104_2024_6826_MOESM1_ESM.pdf]

CLM-Maternal and Infant Health

Start of Block: Screening

Q1
Cannabis **L**egalization in **M**ichigan-Maternal and Infant Health
(CLM)

Welcome ....

Click on the arrow below if you agree to take part in our study.

Page Break

*

Q2 Please enter your study ID.

____

Page Break

Q3 What is your age in **years**?

____

Skip To: End of Block If Condition: What is your age in years? Is Less Than 21. Skip To: End of Block.
Skip To: End of Block If Condition: What is your age in years? Is Greater Than 35. Skip To: End of Block.

Q4 Are you currently pregnant?

- ○ Yes (1)
- ○ No (2)
- ○ I do not know (3)
- ○ I do not wish to answer this question (4)

End of Block: Screening

Start of Block: Baseline data

Q5 I have some general questions about your health.
Would you say your health **in general** is .....

- ◯ Excellent (1)
- ◯ Very good (2)
- ◯ Good (3)
- ◯ Fair (4)
- ◯ Poor (5)
- ◯ I do not know (6)
- ◯ I do not wish to answer this question (7)

Q6 Moderate-intensity sports, fitness or recreational activities cause small increases in breathing or heart rate and is done for **at least 10 minutes** continuously.
In a typical week, on how many days do you do moderate-intensity sports, fitness or recreational activities?

▼ 0 days (1) ... I do not wish to answer this question (10)

Page Break

Q7 The next set of questions are about recent health during the **past 30 days.**

Thinking about your **physical health**, which includes physical illness and injury, for how many days during the past 30 days was your physical health **not good**?

▼ 0 days (1) ... I do not wish to answer this question (33)

Q8 Now thinking about your **mental health**, which includes stress, depression, and problems with emotions, for how many days during the past 30 days was your mental health **not good**?

▼ 0 days (1) ... I do not wish to answer this question (33)

Page Break

Q9 During the past 30 days, for about how many days did poor physical or mental health keep you from doing your usual activities, such as self-care, work, school or recreation?

▼ 0 days (1) ... I do not wish to answer this question (33)

Q10 During the **past 30 days**, for about how many days have you **felt worried, tense, or anxious**?

▼ 0 days (1) ... I do not wish to answer this question (33)

Page Break

Q11 Did you have a **head cold or chest cold** that started during the **past 30 days**?

- ◯ Yes (1)
- ◯ No (2)
- ◯ I do not know (3)
- ◯ I do not wish to answer this question (4)

Display This Question:

If Did you have a head cold or chest cold that started during the past 30 days? = Yes

Q12 During the **past 30 days**, for about how many **days** did you have a **head cold or chest cold**?

▼ 1 day (1) ... I do not wish to answer this question (32)

Q13 Did you have **flu, pneumonia, or ear infections** that started during the **past 30 days**?

- ◯ Yes (1)
- ◯ No (2)
- ◯ I do not know (3)
- ◯ I do not wish to answer this question (4)

Display This Question:

If Did you have flu, pneumonia, or ear infections that started during the past 30 days? = Yes

Q14 During the **past 30 days**, for about how many **days** did you have flu, pneumonia, or ear infections?

▼ 1 day (1) ... I do not wish to answer this question (32)

Page Break

Q15 Just **before you got pregnant**, about how much did you weigh?

- ◯ Weight in pounds (1) ____
- ◯ Weight in Kilograms (2) ____
- ◯ I do not know (3)
- ◯ I do not wish to answer this question (4)

Q16 How tall are you without shoes?

- ◯ Feet and inches (1) ____
- ◯ Meters (2) ____
- ◯ I do not know (3)
- ◯ I do not wish to answer this question (4)

Page Break

Q17 Thinking back to just **before you got pregnant**, how did you feel about becoming pregnant?

- I wanted to be pregnant sooner (1)
- I wanted to be pregnant later (2)
- I wanted to be pregnant then (3)
- I didn't want to be pregnant then or at any time in the future (4)
- I wasn't sure what I wanted (5)
- I do not wish to answer this question (6)

Q18 During the **30 days before you got pregnant**, how many times a week did you take a multivitamin, prenatal vitamin or a folic acid vitamin?

- I didn’t take a multivitamin, prenatal vitamin, or folic acid vitamin at all (1)
- 1 to 3 times a week (2)
- 4 to 6 times a week (3)
- Every day of the week (4)
- I do not know (5)
- I do not wish to answer this question (6)

Page Break

Q19 How many **months** pregnant are you?

- 1 month (1)
- 2 months (2)
- 3 months (3)
- 4 months (4)
- 5 months (5)
- 6 months (6)
- 7 months (7)
- 8 months (8)
- 9 months (9)
- I do not know (10)
- I do not wish to answer this question (11)

Q20 About how much do you weigh **now**?

- Weight in pounds (1) ____
- Weight in Kilograms (2) ____
- I do not know (3)
- I do not wish to answer this question (4)

Q21 During the **past 30 days**, how many times a week did you take a multivitamin, prenatal vitamin or a folic acid vitamin?

- ◯ I didn’t take a multivitamin, prenatal vitamin, or folic acid vitamin at all (1)
- ◯ 1 to 3 times a week (2)
- ◯ 4 to 6 times a week (3)
- ◯ Every day of the week (4)
- ◯ I do not know (5)
- ◯ I do not wish to answer this question (6)

Page Break

Q22 How many times have you been pregnant?
Be sure to **count all your pregnancies** including current pregnancy, live births, miscarriages, stillbirths, ectopic or tubal pregnancies, or abortions.

- ○ 1 (1)
- ○ 2 (2)
- ○ 3 (3)
- ○ 4 (4)
- ○ 5 or more (5)
- ○ I do not know (6)
- ○ I do not wish to answer this question (7)

Display This Question:

If How many times have you been pregnant? Be sure to count all your pregnancies including current pr... != 1

Q23 How many of your pregnancies resulted in a **live birth**?

- 0 (1)
- 1 (2)
- 2 (3)
- 3 (4)
- 4 (5)
- 5 or more (6)
- I do not know (7)
- I do not wish to answer this question (8)

Display This Question:

If How many of your pregnancies resulted in a live birth? != 0

And How many times have you been pregnant? Be sure to count all your pregnancies including current pr... != 1

Q24 How many months ago did you have a baby?

- Less than 6 months ago (1)
- 6-12 months ago (2)
- 13-24 months ago (3)
- More than 24 months (4)
- I do not know (5)
- I do not wish to answer this question (6)

Display This Question:

If How many months ago did you have a baby? = Less than 6 months ago

Or How many months ago did you have a baby? = 6-12 months ago

Or How many months ago did you have a baby? = 13-24 months ago

X→

Q25 Are you now breastfeeding a child?

- ◯ Yes (1)
- ◯ No (2)
- ◯ I do not wish to answer this question (3)

Page Break

Display This Question:

If How many times have you been pregnant? Be sure to count all your pregnancies including current pr... != 1

And How many of your pregnancies resulted in a live birth? != 0

Q26 During your past pregnancy/pregnancies, did you give birth to twins/multiples? If so, how many times did you give birth to twins/multiples?

- No (1)
- Yes, I gave birth to 1 set of twins/multiples (2)

Display This Choice:

If How many times have you been pregnant? Be sure to count all your pregnancies including current pr... = 2

Or How many times have you been pregnant? Be sure to count all your pregnancies including current pr... = 3

Or How many times have you been pregnant? Be sure to count all your pregnancies including current pr... = 4

Or How many times have you been pregnant? Be sure to count all your pregnancies including current pr... = 5 or more

- Yes, I gave birth to 2 sets of twins/multiples (3)

Display This Choice:

If How many times have you been pregnant? Be sure to count all your pregnancies including current pr... = 3

Or How many times have you been pregnant? Be sure to count all your pregnancies including current pr... = 4

Or How many times have you been pregnant? Be sure to count all your pregnancies including current pr... = 5 or more

- Yes, I gave birth to 3 or more sets of twins/multiples (4)
- I do not know (5)
- I do not wish to answer this question (6)

Display This Question:

If How many times have you been pregnant? Be sure to count all your pregnancies including current pr... != 1

Q27 A miscarriage is the spontaneous loss of baby **before the 20th week** of pregnancy.

During your **past pregnancy/pregnancies**, did you have a **miscarriage**(s)? If so, how many miscarriages did you have?

- ○ No (1)
- ○ Yes, I had 1 miscarriage (2)
- ○ Yes, I had 2 miscarriages (3)
- ○ Yes, I had 3 or more miscarriages (4)
- ○ I do not know (5)
- ○ I do not wish to answer this question (6)

Display This Question:

If How many times have you been pregnant? Be sure to count all your pregnancies including current pr... != 1

Q28 A stillbirth is the delivery, **after the 20th week** of pregnancy, of a baby who has died.

During your **past pregnancy/pregnancies**, did you have a **stillbirth**(s)? If so, how many stillbirths did you have?

- ◯ No (1)
- ◯ Yes, I had 1 stillbirth (2)
- ◯ Yes, I had 2 stillbirths (3)
- ◯ Yes, I had 3 or more stillbirths (4)
- ◯ I do not know (5)
- ◯ I do not wish to answer this question (6)

Display This Question:

If How many times have you been pregnant? Be sure to count all your pregnancies including current pr... != 1

X→

Q29 Ectopic pregnancy is when a fertilized egg grows outside the uterus. In more than 90% of cases, the egg implants in a Fallopian tube (tubal pregnancy).

During you **past pregnancy/pregnancies**, did you have an **ectopic pregnancy**? If so, how many ectopic pregnancies did you have?

- ◯ No (1)
- ◯ Yes, I had 1 ectopic pregnancy (2)
- ◯ Yes, I had 2 ectopic pregnancies (3)
- ◯ Yes, I had 3 or more ectopic pregnancies (4)
- ◯ I do not know (5)
- ◯ I do not wish to answer this question (6)

Display This Question:

If How many times have you been pregnant? Be sure to count all your pregnancies including current pr... != 1

Q30 During your **past pregnancy/pregnancies**, did you have a **cesarean section (C-section)**? If so, how many C-sections did you have?

- No (1)
- Yes, I had 1 C-section (2)
- Yes, I had 2 C-sections (3)
- Yes, I had 3 or more C-sections (4)
- I do not know (5)
- I do not wish to answer this question (6)

Page Break

Display This Question:

If How many times have you been pregnant? Be sure to count all your pregnancies including current pr... != 1

And How many of your pregnancies resulted in a live birth? != 0

Q31 How much did your last child weigh at birth?

- ○ Birth weight in pounds (1) ____
- ○ Birth weight in kilograms (2) ____
- ○ I do not know (3)
- ○ I do not wish to answer this question (4)

Display This Question:

If How many times have you been pregnant? Be sure to count all your pregnancies including current pr... != 1

And How many of your pregnancies resulted in a live birth? != 0

Q32 At how many weeks of gestation was your last child delivered?

- ○ Gestational age in weeks (1) ____
- ○ I do not know (2)
- ○ I do not wish to answer this question (3)

Page Break

Display This Question:

If How many times have you been pregnant? Be sure to count all your pregnancies including current pr... != 1

Q33 **During your last pregnancy**, did you experience nausea and/or vomiting?

- Yes (1)
- No (2)
- I do not know (3)
- I do not wish to answer this question (4)

Display This Question:

If During your last pregnancy, did you experience nausea and/or vomiting? = Yes

Q34 Because of your nausea and/or vomiting **during your last pregnancy**, have you ever been told by a doctor to take **prescribed medicine**?

- Yes (1)
- No (2)
- I do not know (3)
- I do not wish to answer this question (4)

Display This Question:

If During your last pregnancy, did you experience nausea and/or vomiting? = Yes

Q35 Did you experience weight loss as a result of your nausea and/or vomiting **during your last pregnancy**?

- Yes (1)
- No (2)
- I do not know (3)
- I do not wish to answer this question (4)

Page Break

Q36 In the **past 30 days**, have you used or taken **medication for which a prescription is needed**?
If yes, please list the **prescription medications** you have used or taken in the past 30 days.

- ○ Yes (1) ____
- ○ No (2)
- ○ I do not know (3)
- ○ I do not wish to answer this question (4)

Q37 In the **past 30 days**, have you used or taken **over-the-counter medication**, **for which a prescription was NOT needed**?
If yes, please list the **over-the-counter medications** you have used or taken in the past 30 days.

- ○ Yes (1) ____
- ○ No (2)
- ○ I do not know (3)
- ○ I do not wish to answer this question (4)

Page Break

Q38 These questions are about the use of tobacco products. The first questions are about cigarettes only.

Have you ever smoked part or all of a cigarette, even once?

- ◯ Yes (1)
- ◯ No (2)
- ◯ I do not know (3)
- ◯ I do not wish to answer this question (4)

Display This Question:

If These questions are about the use of tobacco products. The first questions are about cigarettes o... = Yes

Q39 How long has it been since you last smoked part or all of a cigarette?

- ◯ Within the past 30 days (1)
- ◯ More than 30 days ago but within the past 3 months (2)
- ◯ More than 3 months ago but within the past 6 months (3)
- ◯ More than 6 months ago but within the last 12 months (4)
- ◯ More than 12 months ago (5)
- ◯ I do not know (6)
- ◯ I do not wish to answer this question (7)

Display This Question:

If How long has it been since you last smoked part or all of a cigarette? = Within the past 30 days

Q40 During the past 30 days, on how many days did you **smoke part or all of a c**igarette?

▼ 1 day (1) ... I do not wish to answer this question (32)

Display This Question:

If During the past 30 days, on how many days did you smoke part or all of a cigarette? = I do not know

Or During the past 30 days, on how many days did you smoke part or all of a cigarette? = I do not wish to answer this question

Q41 During the **past 30 days**, what is **your best estimate** of the number of **days you smoked part or all of a cigarette** ?

- ○ 1 to 4 days (1)
- ○ 5 to 10 days (2)
- ○ 11 to 18 days (3)
- ○ 19 to 29 days (4)
- ○ All 30 days (5)
- ○ I do not know (6)
- ○ I do not wish to answer this question (7)

Display This Question:

If How long has it been since you last smoked part or all of a cigarette? = Within the past 30 days

Q42 On the days you **smoked cigarettes** during the **past 30 days**, **how many** cigarettes did you smoke **per day**, on average?

- ◯ Less than one cigarette per day (1)
- ◯ 1 cigarette per day (2)
- ◯ 2 to 5 cigarettes per day (3)
- ◯ 6 to 15 cigarettes per day (about 1⁄2 pack) (4)
- ◯ 16 to 25 cigarettes per day (about 1 pack) (5)
- ◯ 26 to 35 cigarettes per day (about 1 1⁄2 packs) (6)
- ◯ More than 35 cigarettes per day (about 2 packs or more) (7)
- ◯ I do not know (8)
- ◯ I do not wish to answer this question (9)

Page Break

Q43 The following questions ask about using **smokeless tobacco**, such as snuff, dip, chewing tobacco, or snus.

Have you ever used “smokeless” tobacco, even once?

- Yes (1)
- No (2)
- I do not know (3)
- I do not wish to answer this question (4)

Display This Question:

If The following questions ask about using smokeless tobacco, such as snuff, dip, chewing tobacco, o... = Yes

Q44 How long has it been since you last used “smokeless” tobacco?

- Within the past 30 days (1)
- More than 30 days ago but within the past 3 months (2)
- More than 3 months ago but within the past 6 months (3)
- More than 6 months ago but within the last 12 months (4)
- More than 12 months ago (5)
- I do not know (6)
- I do not wish to answer this question (7)

Display This Question:

If How long has it been since you last used “smokeless” tobacco? = Within the past 30 days

Q45 During the past 30 days, on how many days did you use smokeless tobacco?

▼ 1 day (1) ... I do not wish to answer this question (32)

Page Break

Q46 The following questions ask about smoking other tobacco products such as pipes, cigars, little cigars or cigarillos, water pipes, hookahs, or e-cigarettes?   Have you **ever smoked other tobacco products**, even once?

- Yes (1)
- No (2)
- I do not know (3)
- I do not wish to answer this question (4)

Display This Question:

If The following questions ask about smoking other tobacco products such as pipes, cigars, little ci... = Yes

Q47 How long has it been since you **last smoked other tobacco products** such as pipes, cigars, little cigars or cigarillos, water pipes, hookahs, or e-cigarettes?

- Within the past 30 days (1)
- More than 30 days ago but within the past 3 months (2)
- More than 3 months ago but within the past 6 months (3)
- More than 6 months ago but within the last 12 months (4)
- More than 12 months ago (5)
- I do not know (6)
- I do not wish to answer this question (7)

Display This Question:

If How long has it been since you last smoked other tobacco products such as pipes, cigars, little c... = Within the past 30 days

X→

Q48 During the past 30 days, on how many days did you **smoke** other tobacco products such as pipes, cigars, little cigars or cigarillos, water pipes, hookahs, or e-cigarettes?

▼ 1 day (1) ... I do not wish to answer this question (32)

Page Break

Q49 These questions are about drinks of alcoholic beverages. Throughout these questions, by a “drink,” we mean a can or bottle of beer, a glass of wine or a wine cooler, a shot of liquor, or a mixed drink with liquor in it. We are not asking about times when you only had a sip or two from a drink. Have you ever, even once, had a drink of any type of alcoholic beverage? Please do not include times when you only had a sip or two from a drink

- Yes (1)
- No (2)
- I do not know (3)
- I do not wish to answer this question (4)

Display This Question:

If These questions are about drinks of alcoholic beverages. Throughout these questions, by a “drink,... = Yes

Q50 How long has it been since you **last had a drink** of any type of alcoholic beverage? Please do not include times when you only had a sip or two from a drink

- Within the past 30 days (1)
- More than 30 days ago but within the past 3 months (2)
- More than 3 months ago but within the past 6 months (3)
- More than 6 months ago but within the last 12 months (4)
- More than 12 months ago (5)
- I do not know (6)
- I do not wish to answer this question (7)

Display This Question:

If How long has it been since you last had a drink of any type of alcoholic beverage? Please do not... = Within the past 30 days

Q51 During the past 30 days, on how many days did you have **a drink** of any type of alcoholic beverage? Please do not include times when you only had a sip or two from a drink

▼ 1 day (1) ... I do not wish to answer this question (32)

Display This Question:

If During the past 30 days, on how many days did you have a drink of any type of alcoholic beverage?... = I do not know

Or During the past 30 days, on how many days did you have a drink of any type of alcoholic beverage?... = I do not wish to answer this question

Q52 During the **past 30 days**, what is your **best estimate** of the number of **days you had a drink** of any type of alcoholic beverage?

- 1 to 4 days (1)
- 5 to 10 days (2)
- 11 to 18 days (3)
- 19 to 29 days (4)
- All 30 days (5)
- I do not know (6)
- I do not wish to answer this question (7)

Display This Question:

If How long has it been since you last had a drink of any type of alcoholic beverage? Please do not... = Within the past 30 days

Q53 On the days that you drank during the **past 30 days**, have you had **MORE THAN ONE drink** of any type of alcoholic beverage?

- ○ Yes (1)
- ○ No (2)
- ○ I do not know (3)
- ○ I do not wish to answer this question (4)

Page Break

Q54 The next questions are about cannabis (**marijuana)**. Marijuana is also called pot or grass. Marijuana is usually smoked, either in cigarettes, called joints, or in a pipe. It is sometimes cooked in food. Hashish is a form of marijuana that is also called "hash." It is usually smoked in a pipe. Another form of hashish is hash oil.

Please **do not** include cannabidiol (CBD) products such as CBD Gummy Bears or CBD oil, or hemp oil.

Have you **ever**, even once, used **marijuana**?

- Yes (1)
- No (2)
- I do not know (3)
- I do not wish to answer this question (4)

Display This Question:

If The next questions are about cannabis (marijuana). Marijuana is also called pot or grass. Marijua... != Yes

Q55
The answers that people give us about their use of marijuana are **important** to this study's success. We know that this information is personal, but remember your answers will be kept **confidential**.

Please think again about answering this question: Have you **ever**, even once, used **marijuana**?

- Yes (1)
- No (2)
- I do not know (3)
- I do not wish to answer this question (4)

Display This Question:

If The next questions are about cannabis (marijuana). Marijuana is also called pot or grass. Marijua... = Yes

Or The answers that people give us about their use of marijuana are important to this study's succes... = Yes

Q56
How **long** has it been since you **last used marijuana**?

- Within the past 30 days (1)
- More than 30 days ago but within the past 3 months (2)
- More than 3 months ago but within the past 6 months (3)
- More than 6 months ago but within the last 12 months (4)
- More than 12 months ago (5)
- I do not know (6)
- I do not wish to answer this question (7)

Display This Question:

If How long has it been since you last used marijuana? = Within the past 30 days

Q57 During the **past 30 days**, on how many **days** have you used **marijuana** ?

▼ 1 day (1) ... I do not wish to answer this question (32)

Display This Question:

If During the past 30 days, on how many days have you used marijuana ? = I do not know

Or During the past 30 days, on how many days have you used marijuana ? = I do not wish to answer this question

Q58 During the **past 30 days**, what is your **best estimate** of the number of **days you used marijuana**?

- ◯ 1 to 3 days (1)
- ◯ 4 to 10 days (2)
- ◯ 11 to 18 days (3)
- ◯ 19 to 29 days (4)
- ◯ All 30 days (5)
- ◯ I do not know (6)
- ◯ I do not wish to answer this question (7)

Display This Question:

If How long has it been since you last used marijuana? = Within the past 30 days

X→

Q59 During the days that you used **marijuana** in the past 30 days, **how many** would you usually use **in a day**?

- ◯ 1 per day (1)
- ◯ 2 per day (2)
- ◯ 3-5 per day (3)
- ◯ 6 or more per day (4)
- ◯ I do not know (5)
- ◯ I do not wish to answer this question (6)

Page Break

Display This Question:

If The next questions are about cannabis (marijuana). Marijuana is also called pot or grass. Marijua... = Yes

Or The answers that people give us about their use of marijuana are important to this study's succes... = Yes

X→

Q60 How **old** were you the **first time** you used **marijuana**?

- ○ Age in years (1) ____
- ○ I do not know (2)
- ○ I do not wish to answer this question (3)

Display This Question:

If How long has it been since you last used marijuana? = Within the past 30 days

X→

Q61
Was any of your **marijuana** use in the **past 30 days** recommended by a doctor or other health care professional?

- ○ Yes (1)
- ○ No (2)
- ○ I do not know (3)
- ○ I do not wish to answer this question (4)

Display This Question:

If How long has it been since you last used marijuana? = Within the past 30 days

X→

Q62 During the **past 30 days**, how did you use **marijuana**? Select all that apply.

- ▢ Smoked a cigar with marijuana in it, such as a blunt (1)
- ▢ Smoke it (such as a joint, bong, or pipe) (2)
- ▢ Eat it (such as in brownies, cakes, cookies, or candy) (3)
- ▢ Drink it (such as in tea, cola, or alcohol) (4)
- ▢ Vaporize it (such as using a vape pen or e-cigarette-like vaporizer) (5)
- ▢ Dab it (such as using butane hash oil, wax, or concentrates) (6)
- ▢ Used it in some other way. Please specify. (7) ____
- ▢ I do not know (8)
- ▢ I do not wish to answer this question (9)

Page Break

Display This Question:

If The next questions are about cannabis (marijuana). Marijuana is also called pot or grass. Marijua... = Yes

Or The answers that people give us about their use of marijuana are important to this study’s succes... = Yes

Q63 In the 3 months before you were pregnant, were you using marijuana for any reason?

- Yes (1)
- No (2)
- I do not know (3)
- I do not wish to answer this question (4)

Display This Question:

If In the 3 months before you were pregnant, were you using marijuana for any reason? = Yes

Q64 You've indicated that you used marijuana in the 3 months before you were pregnant. Was any of your marijuana use recommended by a doctor or health care professional?

- Yes (1)
- No (2)
- I do not know (3)
- I do not wish to answer this question (4)

Display This Question:

If How many times have you been pregnant? Be sure to count all your pregnancies including current pr... != 1

And The next questions are about cannabis (marijuana). Marijuana is also called pot or grass. Marijua... = Yes

Or The answers that people give us about their use of marijuana are important to this study’s succes... = Yes

X→

Q66 During your last pregnancy, did you use marijuana?

- Yes (1)
- No (2)
- I do not know (3)
- I do not wish to answer this question (4)

Display This Question:

If During your last pregnancy, did you use marijuana? = Yes

Q67 During your last pregnancy, did you use marijuana during the first trimester?

- Yes (1)
- No (2)
- I do not know (3)
- I do not wish to answer (4)

Display This Question:

If During your last pregnancy, did you use marijuana? = Yes

Q68 During your last pregnancy, did you use marijuana during the second trimester?

- Yes (1)
- No (2)
- I do not know (3)
- I do not wish to answer (4)

Display This Question:

If During your last pregnancy, did you use marijuana? = Yes

Q69 During your last pregnancy, did you use marijuana during the third trimester?

- Yes (1)
- No (2)
- I do not know (3)
- I do not wish to answer (4)

Page Break

Q70
The next questions are about **cannabidiol** (**CBD**) products such as C**BD Gummy Bears** or **CBD oil**.

Have you **ever**, even once, used **CBD** products?

- Yes (1)
- No (2)
- I do not know (3)
- I do not wish to answer this question (4)

Display This Question:

If The next questions are about cannabidiol (CBD) products such as CBD Gummy Bears or CBD oil. Hav... = Yes

Q71 How old were you the first time you used **CBD products?**

- Age in years (1) ____
- I do not know (2)
- I do not want to answer this question (3)

Display This Question:

If The next questions are about cannabidiol (CBD) products such as CBD Gummy Bears or CBD oil. Hav... = Yes

Q72
How long has it been since you last used CBD products**?**

- ◯ Within the past 30 days (1)
- ◯ More than 30 days ago but within the past 3 months (2)
- ◯ More than 3 months ago but within the past 6 months (3)
- ◯ More than 6 months ago but within the last 12 months (4)
- ◯ More than 12 months ago (5)
- ◯ I do not know (6)
- ◯ I do not wish to answer this question (7)

Display This Question:

If How long has it been since you last used CBD products? = Within the past 30 days

X→

Q73 During the past 30 days, on **how many days** did you use **CBD products**?

▼ 1 day (1) ... I do not wish to answer this question (32)

Page Break

Q74 Have you ever used **cocaine or crack cocaine**?

- Yes (1)
- No (2)
- I do not know (3)
- I do not wish to answer this question (4)

Display This Question:

If Have you ever used cocaine or crack cocaine? = Yes

Q75 How long has it been since you last used cocaine or crack cocaine?

- Within the past 30 days (1)
- More than 30 days ago but within the past 3 months (2)
- More than 3 months ago but within the past 6 months (3)
- More than 6 months ago but within the last 12 months (4)
- More than 12 months ago (5)
- I do not know (6)
- I do not wish to answer this question (7)

Display This Question:

If How long has it been since you last used cocaine or crack cocaine? = Within the past 30 days

Q76 During the **past 30 days**, on how many **days** have you used **cocaine or crack cocaine**?

▼ 1 day (1) ... I do not wish to answer this question (32)

Page Break

Q77 Have you ever used **heroin**?

- Yes (1)
- No (2)
- I do not know (3)
- I do not wish to answer this question (4)

Display This Question:

If Have you ever used heroin? = Yes

Q78 How long has it been since you last used heroin?

- Within the past 30 days (1)
- More than 30 days ago but within the past 3 months (2)
- More than 3 months ago but within the past 6 months (3)
- More than 6 months ago but within the last 12 months (4)
- More than 12 months ago (5)
- I do not know (6)
- I do not wish to answer this question (7)

Display This Question:

If How long has it been since you last used heroin? = Within the past 30 days

Q79 During the **past 30 days**, on how many **days** have you used **heroin**?

▼ 1 day (1) ... I do not wish to answer this question (32)

Page Break

Q80 Have you ever used **methadone or buprenorphine** ?

- ◯ Yes (1)
- ◯ No (2)
- ◯ I do not know (3)
- ◯ I do not wish to answer this question (4)

Display This Question:

If Have you ever used methadone or buprenorphine ? = Yes

Q81 How long has it been since you last used **methadone or buprenorphine** ?

- ◯ Within the past 30 days (1)
- ◯ More than 30 days ago but within the past 3 months (2)
- ◯ More than 3 months ago but within the past 6 months (3)
- ◯ More than 6 months ago but within the last 12 months (4)
- ◯ More than 12 months ago (5)
- ◯ I do not know (6)
- ◯ I do not wish to answer this question (7)

Display This Question:

If How long has it been since you last used methadone or buprenorphine ? = Within the past 30 days

Q82 During the **past 30 days**, on how many **days** have you used **methadone or buprenorphine** ?

▼ 1 day (1) ... I do not wish to answer this question (32)

Page Break

X→

Q83 Have you ever used **methamphetamine**?

◯ Yes (1)

◯ No (2)

◯ I do not know (3)

◯ I do not wish to answer this question (4)

Display This Question:

If Have you ever used methamphetamine? = Yes

X→

Q84 How long has it been since you **last used methamphetamine**?

◯ Within the past 30 days (1)

◯ More than 30 days ago but within the past 3 months (2)

◯ More than 3 months ago but within the past 6 months (3)

◯ More than 6 months ago but within the last 12 months (4)

◯ More than 12 months ago (5)

◯ I do not know (6)

◯ I do not wish to answer this question (7)

Display This Question:

If How long has it been since you last used methamphetamine? = Within the past 30 days

X→

Q85 During the **past 30 days**, on how many **days** have you used **methamphetamine**?

▼ 1 day (1) ... I do not wish to answer this question (32)

Page Break

Q86 Have you ever used **benzodiazepines**?

- Yes (1)
- No (2)
- I do not know (3)
- I do not wish to answer this question (4)

Display This Question:

If Have you ever used benzodiazepines? = Yes

Q87 How long has it been since you **last used benzodiazepines**?

- Within the past 30 days (1)
- More than 30 days ago but within the past 3 months (2)
- More than 3 months ago but within the past 6 months (3)
- More than 6 months ago but within the last 12 months (4)
- More than 12 months ago (5)
- I do not know (6)
- I do not wish to answer this question (7)

Display This Question:

If How long has it been since you last used benzodiazepines? = Within the past 30 days

Q88 During the **past 30 days**, on how many **days** have you used **benzodiazepines**?

▼ 1 day (1) ... I do not wish to answer this question (32)

Page Break

Q89

These next questions are about any use of **prescription pain relievers**. Please **<u>do not include</u>** “over-the-counter” pain relievers such as aspirin, Tylenol, Advil, or Aleve..

Have you ever, even once, used **any** prescription pain reliever **in any way a doctor did not direct you to use it?**

This includes using it without a prescription of your own, using it in greater amounts, more often, or longer than you were told to take it or using it in any other way a doctor did not direct you to use it such as to feel good or get high?

- ◯ Yes (1)
- ◯ No (2)
- ◯ I do not know (3)
- ◯ I do not wish to answer this question (4)

Display This Question:

If These next questions are about any use of prescription pain relievers. Please do not include “ove... = Yes

Q90 How long has it been since you last used **any prescription pain reliever** in any way a doctor did not direct you to use it?

- ◯ Within the past 30 days (1)
- ◯ More than 30 days ago but within the past 3 months (2)
- ◯ More than 3 months ago but within the past 6 months (3)
- ◯ More than 6 months ago but within the last 12 months (4)
- ◯ More than 12 months ago (5)
- ◯ I do not know (6)
- ◯ I do not wish to answer this question (7)

Display This Question:

If How long has it been since you last used any prescription pain reliever in any way a doctor did n... = Within the past 30 days

X→

Q91 During the **past 30 days**, on how many **days** have you used any prescription pain reliever in any way a doctor did not direct you to use it?

▼ 1 day (1) ... I do not wish to answer this question (32)

Page Break

Q92 Are you of Hispanic, Latino, or Spanish origin or descent?

- ○ Yes (1)
- ○ No (2)
- ○ I do not wish to answer this question (3)

Q93 Which of these groups describes you? You can select more than one group.

- ▢ White (1)
- ▢ Black or African American (2)
- ▢ American Indian or Alaska Native (3)
- ▢ Asian (4)
- ▢ Native Hawaiian or Pacific Islander (5)
- ▢ Other (Please specify) (6) ____
- ▢ I do not wish to answer this question (7)

Page Break

Q94
What is the highest grade of school you have **completed**?

- Less than high school, no diploma (1)
- High school graduate (high school diploma or equivalent including GED) (2)
- Some college credit but no degree (3)
- Associate degree (for example AA or AS) (4)
- Bachelor's degree (for example BA or BS) (5)
- Graduate or professional degree beyond Bachelor's degree (For example Master's degree, doctorate degree, PhD, MD, JD, PharmD, DVM, DDS) (6)
- I do not wish to answer this question (7)

Q95 Information about income is **important** for the study. Please indicate the answer that includes your entire household income in (previous year) before taxes.

- Less than $25,000 (1)
- $25,000 to $49,999 (2)
- $50,000 to $74,999 (3)
- $75,000 to $99,999 (4)
- $100,000 to $149,999 (5)
- $150,000 or more (6)
- I do not wish to answer this question (7)

Q96 Are you covered by.........................?

- Health insurance obtained through employment (1)
- Health insurance purchased directly (2)
- Government programs like Medicaid that help pay my medical bills (3)
- I am not covered by health insurance (4)
- I do not know (5)
- I do not wish to answer this question (6)

Page Break

Q97 Are you **now**......................?

- ◯ Married (1)
- ◯ Widowed (2)
- ◯ Divorced (3)
- ◯ Separated (4)
- ◯ Never married (5)
- ◯ Other (please specify) (6) ____
- ◯ I do not wish to answer this question (7)

Q98 Altogether, how many people live **now** in your household, including yourself?

- 1 (1)
- 2 (2)
- 3 (3)
- 4 (4)
- 5 (5)
- 6 (6)
- 7 (7)
- 8 (8)
- 9 (9)
- 10 or more (10)
- I do not wish to answer this question (11)

Page Break

Display This Question:

If Altogether, how many people live now in your household, including yourself? != 1

Q99

How many **children under the age of 18** live in your household?

- 0 (1)
- 1 (2)
- 2 (3)
- 3 (4)
- 4 (5)
- 5 or more (6)
- I do not know (7)
- I do not wish to answer this question (8)

Display This Question:

If How many children under the age of 18 live in your household? != 0

And Altogether, how many people live now in your household, including yourself? != 1

Q100
How many **children under the age of 6** live in your household?

- ○ 0 (1)
- ○ 1 (2)
- ○ 2 (3)
- ○ 3 (4)
- ○ 4 (5)
- ○ 5 or more (6)
- ○ I do not know (7)
- ○ I do not wish to answer this question (8)

Display This Question:

If How many children under the age of 6 live in your household? != 0

And How many children under the age of 18 live in your household? != 0

And Altogether, how many people live now in your household, including yourself? != 1

X→

Q101
How many **children under the age of 2** live in your household?

- ○ 0 (1)
- ○ 1 (2)
- ○ 2 (3)
- ○ 3 (4)
- ○ 4 (5)
- ○ 5 or more (6)
- ○ I do not know (7)
- ○ I do not wish to answer this question (8)

Page Break

Q102
We are interested in your opinion about the effects of using **marijuana**.

How much do women risk harming themselves physically and in other ways when they use marijuana once a month, **regardless of whether or not pregnant**?

- ○ No risk (1)
- ○ Slight risk (2)
- ○ Moderate risk (3)
- ○ Great risk (4)
- ○ I do not know (5)
- ○ I do not wish to answer this question (6)

Q103
How much do women risk harming themselves physically and in other ways when they use marijuana once or twice a week, **regardless of whether or not pregnant**?

- ○ No risk (1)
- ○ Slight risk (2)
- ○ Moderate risk (3)
- ○ Great risk (4)
- ○ I do not know (5)
- ○ I do not wish to answer this question (6)

Page Break

Q104 How much do **pregnant women** risk harming **themselves** physically and in other ways when they use marijuana once a month?

- No risk (1)
- Slight risk (2)
- Moderate risk (3)
- Great risk (4)
- I do not know (5)
- I do not wish to answer this question (6)

Q105 How much do **pregnant women** risk harming **themselves** physically and in other ways when they use marijuana once or twice a week?

- No risk (1)
- Slight risk (2)
- Moderate risk (3)
- Great risk (4)
- I do not know (5)
- I do not wish to answer this question (6)

Page Break

Q106 Can marijuana use once a month during pregnancy **harm the baby**?

- No risk (1)
- Slight risk (2)
- Moderate risk (3)
- Great risk (4)
- I do not know (5)
- I do not wish to answer this question (6)

Q107 Can marijuana use once or twice a week during pregnancy **harm the baby**?

- No risk (1)
- Slight risk (2)
- Moderate risk (3)
- Great risk (4)
- I do not know (5)
- I do not wish to answer this question (6)

Q108 Can marijuana use while breastfeeding harm the baby?

- No risk (1)
- Slight risk (2)
- Moderate risk (3)
- Great risk (4)
- I do not know (5)
- I do not wish to answer this question (6)

End of Block: Baseline data

Start of Block: Morning sickness

Q109 During your current pregnancy, have you experienced nausea?

- Yes (1)
- No (2)
- I do not know (3)
- I do not wish to answer this question (4)

Skip To: Q110 If During your current pregnancy, have you experienced nausea? = Yes
Skip To: Q112 If During your current pregnancy, have you experienced nausea? = No
Skip To: Q112 If During your current pregnancy, have you experienced nausea? = I do not know
Skip To: Q112 If During your current pregnancy, have you experienced nausea? = I do not wish to answer this question

Q110 On average, in a day, for how long do you feel nauseated or sick to your stomach?

- ◯ Not at all (1)
- ◯ Less than 1 hour (2)
- ◯ 2 to 3 hours (3)
- ◯ 4 to 5 hours (4)
- ◯ 6 or more hours (5)
- ◯ I do not know (6)
- ◯ I do not wish to answer this question (7)

Display This Question:

If During your current pregnancy, have you experienced nausea? = Yes

X→

Q111 Because of your nausea, have you ever been told by a doctor to take **prescribed medicine**?

- ◯ Yes (1)
- ◯ No (2)
- ◯ I do not know (3)
- ◯ I do not wish to answer this question (4)

Page Break

Q112 During your current pregnancy, have you experienced vomiting?

- Yes (1)
- No (2)
- I do not know (3)
- I do not wish to answer this question (4)

Display This Question:

If During your current pregnancy, have you experienced vomiting? = Yes

Q113 On average, in a day, how many times do you vomit or throw up?

- None (1)
- 1 to 2 times (2)
- 3 to 4 times (3)
- 5 to 6 times (4)
- 7 or more times (5)
- I do not know (6)
- I do not wish to answer this question (7)

Display This Question:

If During your current pregnancy, have you experienced vomiting? = Yes

Q114 Because of your vomiting, have you ever been told by a doctor to take **prescribed medicine**?

- Yes (1)
- No (2)
- I do not know (3)
- I do not wish to answer this question (4)

Q115 During your current pregnancy, have you experienced retching or dry heaves without bringing anything up?

- Yes (1)
- No (2)
- I do not know (3)
- I do not wish to answer this question (4)

Display This Question:

If During your current pregnancy, have you experienced retching or dry heaves without bringing anyth... = Yes

X→

Q116 On average, in a day, how many times do you have retching or dry heaves without bringing anything up?

- None (1)
- 1 to 2 times (2)
- 3 to 4 times (3)
- 5 to 6 times (4)
- 7 or more times (5)
- I do not know (6)
- I do not wish to answer this question (7)

Display This Question:

If During your current pregnancy, have you experienced retching or dry heaves without bringing anyth... = Yes

Q117 Because of your retching or dry heaves, have you ever been told by a doctor to take prescribed medicine?

- Yes (1)
- No (2)
- I do not know (3)
- I do not wish to answer this question (4)

End of Block: Morning sickness

Start of Block: Cannabis use during current pregnancy

X→

Q118 What is the date of the first day of your last menstrual period?

- mm/dd/yyyy (1) ____
- I do not know (2)
- I do not wish to answer this question (3)

Page Break

Display This Question:

If The next questions are about cannabis (marijuana). Marijuana is also called pot or grass. Marijua... = Yes

Or The answers that people give us about their use of marijuana are important to this study's succes... = Yes

Q119 During your current pregnancy, did you use marijuana to relieve stress or anxiety?

- Yes (1)
- No (2)
- I do not know (3)
- I do not wish to answer this question (4)

Display This Question:

If The answers that people give us about their use of marijuana are important to this study's succes... = Yes

Or The next questions are about cannabis (marijuana). Marijuana is also called pot or grass. Marijua... = Yes

Q120 During your current pregnancy, did you use marijuana to relieve symptoms of a chronic condition?

- Yes (1)
- No (2)
- I do not know (3)
- I do not wish to answer this question (4)

Display This Question:

If The next questions are about cannabis (marijuana). Marijuana is also called pot or grass. Marijua... = Yes

Or The answers that people give us about their use of marijuana are important to this study's succes... = Yes

Q121 During your current pregnancy, did you use marijuana to relieve pain?

- Yes (1)
- No (2)
- I do not know (3)
- I do not wish to answer this question (4)

Display This Question:

If The next questions are about cannabis (marijuana). Marijuana is also called pot or grass. Marijua... = Yes

Or The answers that people give us about their use of marijuana are important to this study's succes... = Yes

Q122 During your current pregnancy, did you use marijuana for fun or to relax?

- Yes (1)
- No (2)
- I do not know (3)
- I do not wish to answer this question (4)

Display This Question:

If The next questions are about cannabis (marijuana). Marijuana is also called pot or grass. Marijua... = Yes

Or The answers that people give us about their use of marijuana are important to this study’s succes... = Yes

Q123 Is there another reason you used marijuana?

- Yes. Please specify (1) ____
- No (2)
- I do not know (3)
- I do not wish to answer this question (4)

End of Block: Cannabis use during current pregnancy

Start of Block: Tobacco

Display This Question:

If These questions are about the use of tobacco products. The first questions are about cigarettes o... = Yes

Q124 How **old** were you the **first time** you **smoked** part or all of a **cigarette**?

- Age in years (1) ____
- I do not know (2)
- I do not wish to answer this question (3)

Display This Question:

If The following questions ask about using smokeless tobacco, such as snuff, dip, chewing tobacco, o... = Yes

Q125 How **old** were you the **first time** you used “**smokeless**” tobacco?

- ◯ Age in years (1) ____
- ◯ I do not know (2)
- ◯ I do not wish to answer this question (3)

Display This Question:

If The following questions ask about smoking other tobacco products such as pipes, cigars, little ci... = Yes

Q126 Have you **ever smoked** .... **even once**? Check all that apply.

- ▢ Cigars (1)
- ▢ Pipes (2)
- ▢ Little cigars (3)
- ▢ Cigarillos (4)
- ▢ Water pipes/Hookah (5)
- ▢ E-cigarettes (6)
- ▢ Other. Please specify (7) ____
- ▢ I have never smoked any of these tobacco products (8)
- ▢ I do not know (9)
- ▢ I do not wish to answer this question (10)

Display This Question:

If Have you ever smoked .... even once? Check all that apply. = Cigars

Q127 How **old** were you the **first time** you **smoked** part or all of any type of **cigar**?

- ◯ Age in years (1) ____
- ◯ I do not know (2)
- ◯ I do not wish to answer this question (3)

Display This Question:

If Have you ever smoked .... even once? Check all that apply. = Pipes

Q128 How **old** were you the **first time** you smoked tobacco in a pipe, even once?

- ◯ Age in years (1) ____
- ◯ I do not know (2)
- ◯ I do not wish to answer this question (3)

Display This Question:

If Have you ever smoked .... even once? Check all that apply. = Little cigars

Q129 How **old** were you the **first time** you smoked part or all of any type of **little cigars**, even once?

- ◯ Age in years (1) ____
- ◯ I do not know (2)
- ◯ I do not wish to answer this question (3)

Display This Question:

If Have you ever smoked .... even once? Check all that apply. = Cigarillos

Q130 How **old** were you the **first time** you smoked part or all of any type of **cigarillos**, even once?

- Age in years (1) ____
- I do not know (2)
- I do not wish to answer this question (3)

Display This Question:

If Have you ever smoked .... even once? Check all that apply. = E-cigarettes

Q131 How **old** were you the **first time** you smoked **E-cigarettes**, even once?

- Age in years (1) ____
- I do not know (2)
- I do not wish to answer this question (3)

Display This Question:

If Have you ever smoked .... even once? Check all that apply. = Other. Please specify

Q132 How **old** were you the **first time** you smoked **other tobacco products**, even once?

- Age in years (1) ____
- I do not know (2)
- I do not wish to answer this question (3)

End of Block: Tobacco

Start of Block: Medical questions

Q133
Below is a list of health conditions that you may have had during your **lifetime**.
Please read the list and choose **all of the conditions** that **a physician or other health care professional** has ever told you that you had:

☐ Arthritis (1)

☐ Cancer/malignancy (2)

☐ Diabetes mellitus (3)

☐ Heart condition (4)

☐ High blood cholesterol (5)

☐ Kidney condition (6)

☐ HIV/AIDS (7)

☐ Liver condition (8)

☐ Lung disease (9)

☐ Stroke (10)

☐ Other. Please specify (11) ____

☐ Never had any condition (12)

☐ I do not know (13)

☐ I do not wish to answer this question (14)

Display This Question:

If Below is a list of health conditions that you may have had during your lifetime. Please read the... = Lung disease

Q134
You have indicated that a physician or healthcare professional has told you that you have/had a **lung disease**. What **kind** of lung disease was it?

- ☐ Asthma (1)
- ☐ Chronic bronchitis (2)
- ☐ Emphysema (3)
- ☐ Lung cancer (4)
- ☐ Other. Please specify (5) ____
- ☐ I do not know (6)
- ☐ I do not wish to answer this question (7)

Display This Question:

If Below is a list of health conditions that you may have had during your lifetime. Please read the... = Diabetes mellitus

Q135 You have indicated that a physician or healthcare professional has told you that you have/had **diabetes mellitus**. What **type** of diabetes was it?

- ▢ Prediabetes (1)
- ▢ Type 1 diabetes mellitus (2)
- ▢ Type 2 diabetes mellitus (3)
- ▢ Gestational or pregnancy diabetes (4)
- ▢ Other. Please specify (5) ____
- ▢ I do not know (6)
- ▢ I do not wish to answer this question (7)

Display This Question:

If You have indicated that a physician or healthcare professional has told you that you have/had dia... = Prediabetes

Q136 How **old** were you when **prediabetes** was **first diagnosed**?

- ○ Age in years (1) ____
- ○ I do not know (2)
- ○ I do not wish to answer this question (3)

Display This Question:

If You have indicated that a physician or healthcare professional has told you that you have/had dia... = Type 1 diabetes mellitus

Q137 How **old** were you when **type 1** diabetes was **first diagnosed**?

- ◯ Age in years (1) ____
- ◯ I do not know (2)
- ◯ I do not wish to answer this question (3)

Display This Question:

If You have indicated that a physician or healthcare professional has told you that you have/had dia... = Type 2 diabetes mellitus

Q138 How **old** were you when **type 2** diabetes was **first diagnosed**?

- ◯ Age in years (1) ____
- ◯ I do not know (2)
- ◯ I do not wish to answer this question (3)

Display This Question:

If You have indicated that a physician or healthcare professional has told you that you have/had dia... = Gestational or pregnancy diabetes

Q139 How **old** were you when **gestational/pregnancy diabetes** was **first diagnosed**?

- ◯ Age in years (1) ____
- ◯ I do not know (2)
- ◯ I do not wish to answer this question (3)

Display This Question:

If You have indicated that a physician or healthcare professional has told you that you have/had dia... = Other. Please specify

X→

Q140 How **old** were you when **diabetes** was **first diagnosed**?

- ◯ Age in years (1) ____
- ◯ I do not know (2)
- ◯ I do not wish to answer this question (3)

Display This Question:

If Below is a list of health conditions that you may have had during your lifetime. Please read the... = Diabetes mellitus

X→

Q141 Are you now taking **insulin**?

- ◯ Yes (1)
- ◯ No (2)
- ◯ I do not wish to answer this question (3)

Display This Question:

If Below is a list of health conditions that you may have had during your lifetime. Please read the... = Diabetes mellitus

X→

Q142 Are you now taking diabetic pills to lower your blood sugar? These are sometimes called oral agents or oral hypoglycemic agents.

- ◯ Yes (1)
- ◯ No (2)
- ◯ I do not wish to answer this question (3)

Display This Question:

If Below is a list of health conditions that you may have had during your lifetime. Please read the... = Heart condition

Q143 You have indicated that a physician or healthcare professional has told you that you have/had **heart condition**. What **type** of heart condition was it?

- ☐ Angina/angina pectoris (1)
- ☐ Congestive heart disease (2)
- ☐ Coronary heart disease (3)
- ☐ Myocardial infarction/heart attack (4)
- ☐ Other. Please specify (5) ____
- ☐ I do not know (6)
- ☐ I do not wish to answer this question (7)

Q144 **During your current or past pregnancies**, have you ever been told by a physician or healthcare professional that you have/had high blood pressure or preeclampsia?

- ○ Yes (1)
- ○ No (2)
- ○ I do not know (3)
- ○ I do not wish to answer this question (4)

Display This Question:

If During your current or past pregnancies, have you ever been told by a physician or healthcare pro... = Yes

Q145 **During your current or past pregnancies**, were you prescribed medicine for **high blood pressure or** preeclampsia?

- Yes (1)
- No (2)
- I do not know (3)
- I do not wish to answer this question (4)

Page Break

Q146 **Other than during pregnancy**, have you ever been told by a physician or healthcare professional that you have/had high blood pressure or hypertension?

- Yes (1)
- No (2)
- I do not know (3)
- I do not wish to answer this question (4)

Display This Question:

If Other than during pregnancy, have you ever been told by a physician or healthcare professional th... = Yes

Q147 Are you now taking **prescribed** medicine for **high blood pressure or** hypertension?

- Yes (1)
- No (2)
- I do not know (3)
- I do not wish to answer this question (4)

Page Break

Display This Question:

If Below is a list of health conditions that you may have had during your lifetime. Please read the... = High blood cholesterol

Q148 Are you now taking **prescribed** medicine to lower your blood cholesterol?

- Yes (1)
- No (2)
- I do not know (3)
- I do not wish to answer this question (4)

Display This Question:

If Below is a list of health conditions that you may have had during your lifetime. Please read the... = HIV/AIDS

Q149 You have indicated that a physician or healthcare professional has told you that you have/had **liver condition**. What **kind** of liver condition was it?

- ◯ Hepatitis A (1)
- ◯ Hepatitis B (2)
- ◯ Hepatitis C (3)
- ◯ Fatty liver disease (4)
- ◯ Cirrhosis (5)
- ◯ Liver cancer (6)
- ◯ Other. Please specify (7) ____
- ◯ I do not know (8)
- ◯ I do not wish to answer this question (9)

Display This Question:

If Below is a list of health conditions that you may have had during your lifetime. Please read the... = Cancer/malignancy

Q150 You have indicated that a physician or healthcare professional has told you that you have/had **cancer**. What **kind** of cancer was it?

- ☐ Blood (1)
- ☐ Bone (2)
- ☐ Brain (3)
- ☐ Breast (4)
- ☐ Cervix (cervical) (5)
- ☐ Colon (6)
- ☐ Esophagus (esophageal) (7)
- ☐ Gallbladder (8)
- ☐ Kidney (9)
- ☐ Larynx/ windpipe (10)
- ☐ Leukemia (11)
- ☐ Lymphoma/Hodgkin's disease (12)
- ☐ Melanoma (13)
- ☐ Ovary (ovarian) (14)
- ☐ Pancreas (pancreatic) (15)
- ☐ Rectum (rectal) (16)
- ☐ Skin (non-melanoma) (17)

- [ ] Stomach (18)
- [ ] Thyroid (19)
- [ ] Uterus (uterine) (20)
- [ ] Other. Please specify (21) ____
- [ ] I do not know (22)
- [ ] I do not wish to answer this question (23)

End of Block: Medical questions
