# Supplementary material for: A feasibility study to assess the recruitment and retention of pregnant patients who regularly use cannabis
Source: BMC Res Notes. 2024 Jun 25;17:177. doi: 10.1186/s13104-024-06826-4 (PMC11197186; doi:10.1186/s13104-024-06826-4)
Supplement: Supplementary file 2 — Supplementary Material 2 [file 13104_2024_6826_MOESM2_ESM.pdf]

CLM-Maternal and Infant Health, Second &Third Trimester Follow-up

Start of Block: Study ID

Page Break

Q1 Please enter your study ID.

____

End of Block: Study ID

Start of Block: General Health & Pregnancy

Q2 I have some general questions about your health. Since your **LAST** interview, would you say your **health in general** is.....

- ○ Excellent (1)
- ○ Very good (2)
- ○ Good (3)
- ○ Fair (4)
- ○ Poor (5)
- ○ I do not know (6)
- ○ I do not wish to answer this question (7)

Page Break

Q3 The next set of questions are about recent health during the **past 30 days.**

Thinking about your **physical health**, which includes physical illness and injury, for how many days during the past 30 days was your physical health **not good**?

▼ 0 days (1) ... I do not wish to answer this question (33)

Page Break

Q4 Moderate-intensity sports, fitness, or recreational activities cause small increases in breathing or heart rate and is done for at least 10 minutes continuously.

Since your **LAST** interview, in a typical week, on how many days do you do moderate-intensity sports, fitness or recreational activities?

▼ 0 (1) ... I do not wish to answer this question (10)

Page Break

Q5 Now thinking about your **mental health**, which includes stress, depression, and problems with emotions, for how many days during the past 30 days was your mental health **not good**?

▼ 0 days (1) ... I do not wish to answer this question (33)

Q6 During the **past 30 days**, for about how many days have you felt **worried, tense, or anxious**?

▼ 0 days (1) ... I do not wish to answer this question (33)

Page Break

X→

Q7 During the past 30 days, for about how many days did poor physical or mental health keep you from doing your usual activities, such as self-care, work, school or recreation?

▼ 0 days (1) ... I do not wish to answer this question (33)

Page Break

Q8 Did you have a **head cold or chest cold** that started during the **past 30 days**?

- ◯ Yes (1)
- ◯ No (2)
- ◯ I do not know (3)
- ◯ I do not wish to answer this question (4)

Display This Question:

If Did you have a head cold or chest cold that started during the past 30 days? = Yes

Q9 During the **past 30 days**, for about how many **days** did you have a **head cold or chest cold**?

▼ 1 day (1) ... I do not wish to answer this question (32)

Page Break

Q10 Did you have **flu, pneumonia, or ear infections** that started during the **past 30 days**?

- ◯ Yes (1)
- ◯ No (2)
- ◯ I do not know (3)
- ◯ I do not wish to answer this question (4)

Display This Question:

If Did you have flu, pneumonia, or ear infections that started during the past 30 days? = Yes

Q11 During the **past 30 days**, for about how many **days** did you have flu, pneumonia, or ear infections?

▼ 1 day (1) ... I do not wish to answer this question (32)

Page Break

Q12 How many **months** pregnant are you now?

- 4 (1)
- 5 (2)
- 6 (3)
- 7 (4)
- 8 (5)
- 9 (6)
- Other. Please specify (7) ____

Q13 About how much do you weigh **now**?

- Weight in pounds (1) ____
- Weight in Kilograms (2) ____
- I do not know (3)
- I do not wish to answer this question (4)

Page Break

Q14 During the **past 30 days**, how many times a week did you take a multivitamin, prenatal vitamin or a folic acid vitamin?

- ○ I didn't take a multivitamin, prenatal vitamin, or folic acid vitamin at all (1)
- ○ 1 to 3 times a week (2)
- ○ 4 to 6 times a week (3)
- ○ Every day of the week (4)
- ○ I do not know (5)
- ○ I do not wish to answer this question (6)

Page Break

Q15
Since your **LAST** interview, has a physician or healthcare professional told you that you have gestational diabetes, or pregnancy diabetes?

- ☐ Yes (1)
- ☐ No (2)
- ☐ I do not know (3)
- ☐ I do not wish to answer this question (4)

Q16 Since your **LAST** interview, has a physician or healthcare professional told you that you have high blood pressure during pregnancy, or preeclampsia?

- ○ Yes (1)
- ○ No (2)
- ○ I do not know (3)
- ○ I do not wish to answer this question (4)

End of Block: General Health & Pregnancy

Start of Block: Morning Sickness

Q17 Since your **LAST** interview, have you experienced nausea during your pregnancy?

- Yes (1)
- No (2)
- I do not know (3)
- I do not wish to answer this question (4)

Display This Question:

If Since your LAST interview, have you experienced nausea during your pregnancy? = Yes

X→

Q18 On average, in a day, for how long do you feel nauseated or sick to your stomach?

- Not at all (1)
- 1 hour or less (2)
- 2 tot 3 hours (3)
- 4 to 6 hours (4)
- More than 6 hours (5)
- I do not know (6)
- I do not wish to answer this question (7)

Display This Question:

If Since your LAST interview, have you experienced nausea during your pregnancy? = Yes

X→

Q19 Because of your nausea, have you been told by a doctor to take prescribed medicine?

- Yes (1)
- No (2)
- I do not know (3)
- I do not wish to answer this question (4)

X→

Q20 Since your **LAST** interview, have you experienced vomiting during your pregnancy?

- Yes (1)
- No (2)
- I do not know (3)
- I do not wish to answer this question (4)

Display This Question:

If Since your LAST interview, have you experienced vomiting during your pregnancy? = Yes

X→

Q21 On average, in a day, how many times do you vomit or throw up?

- None (1)
- 1 to 2 times (2)
- 3 to 4 times (3)
- 5 to 6 times (4)
- 7 or more times (5)
- I do not know (6)
- I do not wish to answer this question (7)

Display This Question:

If Since your LAST interview, have you experienced vomiting during your pregnancy? = Yes

Q22 Because of your vomiting, have you been told by a doctor to take prescribed medicine?

- Yes (1)
- No (2)
- I do not know (3)
- I do not wish to answer this question (4)

Q23 Since your **LAST** interview, have you experienced retching or dry heaves without bringing anything up?

- Yes (1)
- No (2)
- I do not know (3)
- I do not wish to answer this question (4)

Display This Question:

If Since your LAST interview, have you experienced retching or dry heaves without bringing anything... = Yes

Q24 On average, in a day, how many times do you have retching or dry heaves without bringing anything up?

- None (1)
- 1 to 2 times (2)
- 3 to 4 times (3)
- 5 to 6 times (4)
- 7 or more times (5)
- I do not know (6)
- I do not wish to answer this question (7)

Display This Question:

If Since your LAST interview, have you experienced retching or dry heaves without bringing anything... = Yes

Q25 Because of your retching or dry heaves, have you been told by a doctor to take prescribed medicine?

- ◯ Yes (1)
- ◯ No (2)
- ◯ I do not know (3)
- ◯ I do not wish to answer this question (4)

End of Block: Morning Sickness

Start of Block: Drug use

Q26 Since your **LAST** interview, have you used or taken medication for which a **prescription** is needed? If yes, please list the prescription medications you have taken since your **LAST** interview.

- ◯ Yes (1) ____
- ◯ No (2)
- ◯ I do not know (3)
- ◯ I do not wish to answer this question (4)

Q27 Since your **LAST** interview, have you used or taken **over-the-counter medication**, for which a prescription was NOT needed? If yes, please list the **over-the-counter medications** you have used or taken since your **LAST** interview.

- ○ Yes (1) ____
- ○ No (2)
- ○ I do not know (3)
- ○ I do not wish to answer this question (4)

Display This Question:

If Since your LAST interview, have you used or taken medication for which a prescription is needed?... = Yes

X→

Q28 In the **past 30 days**, have you used or taken medication for which a **prescription** is needed? If yes, please list the prescription medications you have used or taken in the **past 30 days**.

- ○ Yes (1) ____
- ○ No (2)
- ○ I do not know (3)
- ○ I do not wish to answer this question (4)

Display This Question:

If Since your LAST interview, have you used or taken over-the-counter medication, for which a prescr... = Yes

X→

Q29 In the **past 30 days**, have you used or taken **over-the-counter medication**, for which a prescription was NOT needed? If yes, please list the **over-the-counter medications** you have used or taken in the **past 30 days**.

- ○ Yes (1) ____
- ○ No (2)
- ○ I do not know (3)
- ○ I do not wish to answer this question (4)

Page Break

Q30 These questions are about the use of tobacco products. The first questions are about cigarettes only.

Since your **LAST** interview, have you smoked part or all of a **cigarette**?

- ○ Yes (1)
- ○ No (2)
- ○ I do not know (3)
- ○ I do not wish to answer this question (4)

Skip To: Q33 If These questions are about the use of tobacco products. The first questions are about cigarettes o... = No

Skip To: Q33 If These questions are about the use of tobacco products. The first questions are about cigarettes o... = I do not know

Skip To: Q33 If These questions are about the use of tobacco products. The first questions are about cigarettes o... = I do not wish to answer this question

Q31 During the past 30 days, on how many days did you **smoke part or all of a c**igarette?

▼ 0 days (1) ... I do not wish to answer this question (33)

Skip To: Q33 If During the past 30 days, on how many days did you smoke part or all of a cigarette? = 0 days

Q32 On the days you **smoked cigarettes** during the **past 30 days**, **how many** cigarettes did you smoke **per day**, on average?

- Less than one cigarette per day (1)
- 1 cigarette per day (2)
- 2 to 5 cigarettes per day (3)
- 6 to 15 cigarettes per day (about 1⁄2 pack) (4)
- 16 to 25 cigarettes per day (about 1 pack) (5)
- 26 to 35 cigarettes per day (about 1 1⁄2 packs) (6)
- More than 35 cigarettes per day (about 2 packs or more) (7)
- I do not know (8)
- I do not wish to answer this question (9)

Q33 The following questions ask about using **smokeless tobacco**, such as snuff, dip, chewing tobacco, or snus.

Since your LAST interview, have you used “smokeless” tobacco, even once?

- Yes (1)
- No (2)
- I do not know (3)
- I do not wish to answer this question (4)

Display This Question:

If The following questions ask about using smokeless tobacco, such as snuff, dip, chewing tobacco, o... = Yes

Q34 During the past 30 days, on how many days did you use smokeless tobacco?

▼ 0 days (1) ... I do not wish to answer this question (33)

Q35 The following questions ask about smoking other tobacco products such as pipes, cigars, little cigars or cigarillos, water pipes, hookahs, or e-cigarettes.   Since your LAST interview, have you **ever smoked other tobacco products**, even once?

◯ Yes (1)

◯ No (2)

◯ I do not know (3)

◯ I do not wish to answer this question (4)

Display This Question:

If The following questions ask about smoking other tobacco products such as pipes, cigars, little ci... = Yes

Q36 During the past 30 days, on how many days did you **smoke** other tobacco products such as pipes, cigars, little cigars or cigarillos, water pipes, hookahs, or e-cigarettes?

▼ 0 days (1) ... I do not wish to answer this question (33)

Page Break

Q37 Since your **LAST** interview, have you had a drink of any type of **alcoholic beverage**? Please do not include times when you only had a sip or two from a drink.

- Yes (1)
- No (2)
- I do not know (3)
- I do not wish to answer this question (4)

Display This Question:

If Since your LAST interview, have you had a drink of any type of alcoholic beverage? Please do not... = Yes

Q38 During the **past 30 days**, on how many days did you **have a drink of any type of alcoholic beverage**?

▼ 0 days (1) ... I do not wish to answer this question (33)

Page Break

Q39
Since your **LAST** interview, have you used **marijuana**?

- Yes (1)
- No (2)
- I do not know (3)
- I do not wish to answer this question (4)

Skip To: Q45 If Since your LAST interview, have you used marijuana? = No
Skip To: Q45 If Since your LAST interview, have you used marijuana? = I do not know
Skip To: Q45 If Since your LAST interview, have you used marijuana? = I do not wish to answer this question

---

Q40
Was any of your **marijuana** use recommended by a doctor or other health care professional?

- Yes (1)
- No (2)
- I do not know (3)
- I do not wish to answer this question (4)

Q41 During the **past 30 days**, on how many **days** have you used **marijuana** ?

▼ 0 days (1) ... I do not wish to answer this question (33)

Skip To: Q45 If During the past 30 days, on how many days have you used marijuana ? = 0 days

---

Display This Question:

If During the past 30 days, on how many days have you used marijuana ? = I do not wish to answer this question

Or During the past 30 days, on how many days have you used marijuana ? = I do not know

Q42 During the **past 30 days**, what is your **best estimate** of the number of **days you used marijuana**?

- 0 days (1)
- 1 or 4 days (2)
- 5 to 10 days (3)
- 11 to 18 days (4)
- 19 to 29 days (5)
- All 30 days (6)
- I do not know (7)
- I do not wish to answer this question (8)

Skip To: Q45 If During the past 30 days, what is your best estimate of the number of days you used marijuana? = 0 days

Skip To: Q45 If During the past 30 days, what is your best estimate of the number of days you used marijuana? = I do not know

Skip To: Q45 If During the past 30 days, what is your best estimate of the number of days you used marijuana? = I do not wish to answer this question

Q43 During the **past 30 days**, how did you use **marijuana**? Select all that apply

- ☐ Smoked a cigar with marijuana in it, such as a blunt (1)
- ☐ Smoke it (such as a joint, bong, or pipe) (2)
- ☐ Eat it (such as in brownies, cakes, cookies, or candy) (3)
- ☐ Drink it (such as in tea, cola, or alcohol) (4)
- ☐ Vaporize it (such as using a vape pen or e-cigarette-like vaporizer) (5)
- ☐ Dab it (such as using butane hash oil, wax, or concentrates) (6)
- ☐ Used it in some other way. Please specify. (7) ________________
- ☐ I do not know (8)
- ☐ I do not wish to answer this question (9)

Q44 During the days that you used **marijuana** in the past 30 days, **how many** would you usually use **in a day**?

- ○ 1 per day (1)
- ○ 2 per day (2)
- ○ 3-5 per day (3)
- ○ 6 or more per day (4)
- ○ I do not know (5)
- ○ I do not wish to answer this question (6)

Page Break

Q45 The next questions are about **cannabidiol (CBD) products** such as **CBD Gummy Bears or CBD oil**.

Since your **LAST** interview, have you used **CBD products**?

- Yes (1)
- No (2)
- I do not know (3)
- I do not wish to answer this question (4)

Display This Question:

If The next questions are about cannabidiol (CBD) products such as CBD Gummy Bears or CBD oil. Since... = Yes

Q46 During the **past 30 days**, on how many days did you use **CBD products**?

▼ 0 days (1) ... I do not wish to answer this question (33)

Q47 Since your **LAST** interview, have you used cocaine or crack cocaine?

- Yes (1)
- No (2)
- I do not know (3)
- I do not wish to answer this question (4)

Display This Question:

If Since your LAST interview, have you used cocaine or crack cocaine? = Yes

Q48 During the **past 30 days**, on how many days have you used **cocaine or crack cocaine**?

▼ 0 days (1) ... I do not wish to answer this question (33)

Q49 Since your **LAST** interview, have you used heroin?

- ○ Yes (1)
- ○ No (2)
- ○ I do not know (3)
- ○ I do not wish to answer this question (4)

Display This Question:

If Since your LAST interview, have you used heroin? = Yes

Q50 During the **past 30 days**, on how many days have you used **heroin**?

▼ 0 days (1) ... I do not wish to answer this question (33)

Q51 Since your **LAST** interview, have you used **methadone or buprenorphine?**

- Yes (1)
- No (2)
- I do not know (3)
- I do not wish to answer this question (4)

Display This Question:

If Since your LAST interview, have you used methadone or buprenorphine? = Yes

X→

Q52 During the **past 30 days**, on how many days have you used **methadone or buprenorphine** ?

▼ 0 days (1) ... I do not wish to answer this question (33)

X→

Q53 Since your **LAST** interview, have you used **methamphetamine**?

- Yes (1)
- No (2)
- I do not know (3)
- I do not wish to answer this question (4)

Display This Question:

If Since your LAST interview, have you used methamphetamine? = Yes

X→

Q54 During the **past 30 days**, on how many days have you used **methamphetamine**?

▼ 0 days (1) ... I do not wish to answer this question (33)

X→

Q55 Since you **LAST** interview, have you used **benzodiazepines**?

- ◯ Yes (1)
- ◯ No (2)
- ◯ I do not know (3)
- ◯ I do not wish to answer this question (4)

Display This Question:

If Since you LAST interview, have you used benzodiazepines? = Yes

X→

Q56 During the **past 30 days**, on how many days have you used **benzodiazepines**?

▼ 0 days (1) ... I do not wish to answer this question (33)

X→

Q57 Since your **LAST** interview, have you used **any prescription pain reliever** in any way a doctor did not direct you to use it, including using it without a prescription of your own; using it in greater amounts, more often, or longer than you were told to take it; or using it in any other way a doctor did not direct you to use it such as to feel good or get high?

- ◯ Yes (1)
- ◯ No (2)
- ◯ I do not know (3)
- ◯ I do not wish to answer this question (4)

Display This Question:

If Since your LAST interview, have you used any prescription pain reliever in any way a doctor did n... = Yes

X→

Q58 During the **past 30 days**, on how many days have you used any prescription pain reliever in any way a doctor did not direct you to use it?

▼ 0 days (1) ... I do not wish to answer this question (33)

End of Block: Drug use

Start of Block: Risk Perception

X→

Q59
During your **LAST** interview, we asked about your opinion regarding the effects of using **marijuana**.

What is your current opinion: How much do women risk harming themselves physically and in other ways when they use marijuana once a month, **regardless of whether or not pregnant**?

- No risk (1)
- Slight risk (2)
- Moderate risk (3)
- Great risk (4)
- I do not know (5)
- I do not wish to answer this question (6)

Q60
What is your current opinion: How much do women risk harming themselves physically and in other ways when they use marijuana once or twice a week, **regardless of whether or not pregnant**?

- No risk (1)
- Slight risk (2)
- Moderate risk (3)
- Great risk (4)
- I do not know (5)
- I do not wish to answer this question (6)

Q61 **What is your current opinion:** How much do **pregnant women** risk harming themselves physically and in other ways when they use marijuana once a month?

- No risk (1)
- Slight risk (2)
- Moderate risk (3)
- Great risk (4)
- I do not know (5)
- I do not wish to answer this question (6)

Q62 **What is your current opinion:** How much do **pregnant women** risk harming themselves physically and in other ways when they use marijuana once or twice a week?

- No risk (1)
- Slight risk (2)
- Moderate risk (3)
- Great risk (4)
- I do not know (5)
- I do not wish to answer this question (6)

Q63 **What is your current opinion:** Can marijuana use once a month during pregnancy **harm the baby**?

- ◯ No risk (1)
- ◯ Slight risk (2)
- ◯ Moderate risk (3)
- ◯ Great risk (4)
- ◯ I do not know (5)
- ◯ I do not wish to answer this question (6)

Q64 **What is your current opinion:** Can marijuana use once or twice a week during pregnancy **harm the baby**?

- ◯ No risk (1)
- ◯ Slight risk (2)
- ◯ Moderate risk (3)
- ◯ Great risk (4)
- ◯ I do not know (5)
- ◯ I do not wish to answer this question (6)

Q65 **What is your current opinion:** Can marijuana use while breastfeeding harm the infant?

- ○ No risk (1)
- ○ Slight risk (2)
- ○ Moderate risk (3)
- ○ Great risk (4)
- ○ I do not know (5)
- ○ I do not wish to answer this question (6)

Page Break

Q66 In the 3 months before you were pregnant, were you using marijuana for any reason?

- Yes (1)
- No (2)
- I do not know (3)
- I do not wish to answer this question (4)

End of Block: Risk Perception

Start of Block: Cannabis use during pregnancy

Q67 Since your **LAST** interview, did you use marijuana to **relieve stress or anxiety** during your pregnancy?

- Yes (1)
- No (2)
- I do not know (3)
- I do not wish to answer this question (4)

Q68 Since your **LAST** interview, did you use marijuana to **relieve symptoms of a chronic condition** during your pregnancy?

- Yes (1)
- No (2)
- I do not know (3)
- I do not wish to answer this question (4)

Q69 Since your **LAST** interview, did you use marijuana to **relieve pain** during your pregnancy?

- ◯ Yes (1)
- ◯ No (2)
- ◯ I do not know (3)
- ◯ I do not wish to answer this question (4)

Q70 Since your **LAST** interview, did you use marijuana **for fun or to relax** during your pregnancy?

- ◯ Yes (1)
- ◯ No (2)
- ◯ I do not know (3)
- ◯ I do not wish to answer this question (4)

Q71 Since your **LAST** interview, is there **another reason** you used marijuana during your pregnancy?

- ◯ Yes. Please specify (1) ____
- ◯ No (2)
- ◯ I do not know (3)
- ◯ I do not wish to answer this question (4)

End of Block: Cannabis use during pregnancy
