# Supplementary material for: A feasibility study to assess the recruitment and retention of pregnant patients who regularly use cannabis
Source: BMC Res Notes. 2024 Jun 25;17:177. doi: 10.1186/s13104-024-06826-4 (PMC11197186; doi:10.1186/s13104-024-06826-4)
Supplement: Supplementary file 3 — Supplementary Material 3 [file 13104_2024_6826_MOESM3_ESM.pdf]

CLM-Maternal and Infant Health, Post-delivery Follow-up

Start of Block: Study ID

Q1 Please enter your study ID.

____

End of Block: Study ID

Start of Block: Drug use

Q2 Since your **LAST** interview, have you used or taken medication for which a **prescription** was needed?

- Yes. Please list your medication below: (1) ____
- No (2)
- I do not know (3)
- I do not wish to answer this question (4)

Q3 Since your **LAST** interview, have you used or taken an **over-the-counter medication**, for which a prescription was **NOT** needed?

- Yes. Please list your medication below: (1) ____
- No (2)
- I do not know (3)
- I do not wish to answer this question (4)

Page Break

Display This Question:

If Since your LAST interview, have you used or taken medication for which a prescription was needed? = Yes. Please list your medication below:

Q4 In the **past 30 days**, have you used or taken medication for which a **prescription** was needed?

- Yes. Please list your medication below: (1) ____
- No (2)
- I do not know (3)
- I do not wish to answer this question (4)

Display This Question:

If Since your LAST interview, have you used or taken an over-the-counter medication, for which a pre... = Yes. Please list your medication below:

Q5 In the **past 30 days**, have you used or taken **over-the-counter medication**, for which a prescription was **NOT** needed?

- Yes. Please list your medication below: (1) ____
- No (2)
- I do not know (3)
- I do not wish to answer this question (5)

Page Break

Q6 These questions are about the use of tobacco products. The first questions are about cigarettes only.
Since your **LAST** interview, have you smoked part or all of a **cigarette**?

- ◯ Yes (1)
- ◯ No (2)
- ◯ I do not know (3)
- ◯ I do not wish to answer this question (4)

Display This Question:

If These questions are about the use of tobacco products. The first questions are about cigarettes o... = Yes

Q7 During the past 30 days, on how many days did you **smoke part or all of a c**igarette?

▼ 0 days (1) ... I do not wish to answer this question (64)

Skip To: Q9 If During the past 30 days, on how many days did you smoke part or all of a cigarette? = 0 days

Skip To: Q9 If During the past 30 days, on how many days did you smoke part or all of a cigarette? = I do not know

Skip To: Q9 If During the past 30 days, on how many days did you smoke part or all of a cigarette? = I do not wish to answer this question

---

Display This Question:

If These questions are about the use of tobacco products. The first questions are about cigarettes o... = Yes

Q8 On the days you **smoked cigarettes** during the **past 30 days**, **how many** cigarettes did you smoke **per day**, on average?

○ Less than one cigarette per day (1)

○ 1 cigarette per day (2)

○ 2 to 5 cigarettes per day (3)

○ 6 to 15 cigarettes per day (about 1⁄2 pack) (4)

○ 16 to 25 cigarettes per day (about 1 pack) (5)

○ 26 to 35 cigarettes per day (about 1 1⁄2 packs) (6)

○ More than 35 cigarettes per day (about 2 packs or more) (7)

○ I do not know (9)

○ I do not wish to answer this question (8)

Page Break

Q9 The following questions ask about using **smokeless tobacco**, such as snuff, dip, chewing tobacco, or snus.

Since your LAST interview, have you used “smokeless” tobacco, even once?

- ◯ Yes (1)
- ◯ No (4)
- ◯ I do not know (5)
- ◯ I do not wish to answer this question (6)

Display This Question:

If The following questions ask about using smokeless tobacco, such as snuff, dip, chewing tobacco, o... = Yes

Q10 During the past 30 days, on how many days did you use smokeless tobacco?

▼ 0 days (1) ... I do not wish to answer this question (64)

Page Break

Q11 The following questions ask about smoking other tobacco products such as pipes, cigars, little cigars or cigarillos, water pipes, hookahs, or e-cigarettes.   Since your LAST interview, have you **ever smoked other tobacco products**, even once?

- Yes (1)
- No (2)
- I do not know (4)
- I do not wish to answer this question (5)

Display This Question:

If The following questions ask about smoking other tobacco products such as pipes, cigars, little ci... = Yes

Q12 During the past 30 days, on how many days did you **smoke** other tobacco products such as pipes, cigars, little cigars or cigarillos, water pipes, hookahs, or e-cigarettes?

▼ 0 days (1) ... I do not wish to answer this question (64)

Page Break

Q13 Since your **LAST** interview, have you had a drink of any type of **alcoholic beverage**? Please do not include times when you only had a sip or two from a drink.

- Yes (1)
- No (2)
- I do not know (3)
- I do not wish to answer this question (4)

Display This Question:

If Since your LAST interview, have you had a drink of any type of alcoholic beverage? Please do not... = Yes

Q14 During the **past 30 days**, on how many days did you **have a drink of any type of alcoholic beverage**?

▼ 0 days (77) ... I do not wish to answer this question (76)

Page Break

Q15
Since your **LAST** interview, have you used **marijuana**?

- ◯ Yes (1)
- ◯ No (2)
- ◯ I do not know (3)
- ◯ I do not wish to answer this question (4)

Skip To: Q21 If Since your LAST interview, have you used marijuana? != Yes

---

Display This Question:

If Since your LAST interview, have you used marijuana? = Yes

Q16 During the **past 30 days**, on how many **days** have you used **marijuana** ?

▼ 0 days (67) ... I do not wish to answer this question (66)

Skip To: Q21 If During the past 30 days, on how many days have you used marijuana ? = 0 days

---

Display This Question:

If During the past 30 days, on how many days have you used marijuana ? = I do not know

Or During the past 30 days, on how many days have you used marijuana ? = I do not wish to answer this question

Q17 During the **past 30 days**, what is your **best estimate** of the number of **days you used marijuana**?

- 0 days (11)
- 1 or 4 days (1)
- 5 to 10 days (4)
- 11 to 18 days (5)
- 19 to 29 days (6)
- All 30 days (8)
- I do not know (9)
- I do not wish to answer this question (10)

Skip To: Q21 If During the past 30 days, what is your best estimate of the number of days you used marijuana? = 0 days

Skip To: Q21 If During the past 30 days, what is your best estimate of the number of days you used marijuana? = I do not know

Skip To: Q21 If During the past 30 days, what is your best estimate of the number of days you used marijuana? = I do not wish to answer this question

Q18 During the days that you used **marijuana** in the past 30 days, **how many** would you usually use **in a day**?

- 1 per day (1)
- 2 per day (4)
- 3-5 per day (5)
- Six or more per day (6)
- I do not know (7)
- I do not wish to answer this question (8)

Q19
Was any of your **marijuana** use in the **past 30 days** recommended by a doctor or other health care professional?

- ○ Yes (1)
- ○ No (2)
- ○ I do not know (4)
- ○ I do not wish to answer this question (5)

Q20 During the **past 30 days**, how did you use **marijuana**?

- ☐ Smoked a cigar with marijuana in it, such as a blunt (1)
- ☐ Smoke it (such as a joint, bong, or pipe) (4)
- ☐ Eat it (such as in brownies, cakes, cookies, or candy) (5)
- ☐ Drink it (such as in tea, cola, or alcohol) (6)
- ☐ Vaporize it (such as using a vape pen or e-cigarette-like vaporizer) (7)
- ☐ Dab it (such as using butane hash oil, wax, or concentrates) (8)
- ☐ Used it in some other way. Please specify. (9) ____
- ☐ I do not know (10)
- ☐ I do not wish to answer this question (11)

Page Break

Q21 The next questions are about **cannabidiol (CBD) products** such as **CBD Gummy Bears or CBD oil**.

Since your **LAST** interview, have you used **CBD products**?

- ◯ Yes (1)
- ◯ No (4)
- ◯ I do not know (5)
- ◯ I do not wish to answer this question (6)

Display This Question:

If The next questions are about cannabidiol (CBD) products such as CBD Gummy Bears or CBD oil. Since... = Yes

Q22 During the **past 30 days**, on how many days did you use **CBD products**?

▼ 0 days (4) ... I do not wish to answer this question (35)

Page Break

Q23 Since your **LAST** interview, have you used cocaine or crack cocaine?

- ◯ Yes (1)
- ◯ No (2)
- ◯ I do not know (3)
- ◯ I do not wish to answer this question (4)

Display This Question:

If Since your LAST interview, have you used cocaine or crack cocaine? = Yes

Q24 During the **past 30 days**, on how many days have you used **cocaine or crack cocaine**?

▼ 0 days (1) ... I do not wish to answer this question (63)

Page Break

Q25 Since your **LAST** interview, have you used heroin?

- ○ Yes (1)
- ○ No (2)
- ○ I do not know (3)
- ○ I do not wish to answer this question (4)

Display This Question:

If Since your LAST interview, have you used heroin? = Yes

Q26 During the **past 30 days**, on how many days have you used **heroin**?

▼ 0 days (1) ... I do not wish to answer this question (63)

Page Break

Q27 Since your **LAST** interview, have you used **methadone or buprenorphine?**

- Yes (1)
- No (2)
- I do not know (3)
- I do not wish to answer this question (4)

Display This Question:

If Since your LAST interview, have you used methadone or buprenorphine? = Yes

Q28 During the **past 30 days**, on how many days have you used **methadone or buprenorphine** ?

▼ 0 days (1) ... I do not wish to answer this question (63)

Page Break

Q29 Since your **LAST** interview, have you used **methamphetamine**?

- ◯ Yes (1)
- ◯ No (2)
- ◯ I do not know (3)
- ◯ I do not wish to answer this question (4)

Display This Question:

If Since your LAST interview, have you used methamphetamine? = Yes

Q30 During the **past 30 days**, on how many days have you used methamphetamine?

▼ 0 days (1) ... I do not wish to answer this question (63)

Page Break

Q31 Since you **LAST** interview, have you used **benzodiazepines**?

- ◯ Yes (1)
- ◯ No (2)
- ◯ I do not know (3)
- ◯ I do not wish to answer this question (4)

Display This Question:

If Since you LAST interview, have you used benzodiazepines? = Yes

Q32 During the **past 30 days**, on how many days have you used benzodiazepines?

▼ 0 days (1) ... I do not wish to answer this question (63)

Page Break

Q33 Since your **LAST** interview, have you used **any prescription pain reliever** in any way a doctor **did not direct you to use it**, including using it **without a prescription of your own**; using it in **greater amounts, more often, or longer** than you were told to take it; or using it in any other way a doctor did not direct you to use it such as to **feel good or get high**?

- ◯ Yes (1)
- ◯ No (2)
- ◯ I do not know (3)
- ◯ I do not wish to answer this question (4)

Display This Question:

If Since your LAST interview, have you used any prescription pain reliever in any way a doctor did n... = Yes

Q34 During the **past 30 days**, on how many days have you used any prescription pain reliever in any way a doctor did not direct you to use it?

▼ 0 days (1) ... I do not wish to answer this question (63)

End of Block: Drug use

Start of Block: General Health & Pregnancy

Q35
Since your **LAST** interview, has a physician or healthcare professional told you that you have gestational diabetes, or pregnancy diabetes?

- ▢ Yes (1)
- ▢ No (2)
- ▢ I do not know (3)
- ▢ I do not wish to answer this question (4)

Q36 Since your **LAST** interview, has a physician or healthcare professional told you that you have high blood pressure during pregnancy, or preeclampsia?

- ◯ Yes (1)
- ◯ No (2)
- ◯ I do not know (3)
- ◯ I do not wish to answer this question (4)

End of Block: General Health & Pregnancy

Start of Block: Risk Perception

Q37
During your **LAST** interview, we asked about your opinion regarding the effects of using **marijuana**.

What is your current opinion: How much do women risk **harming themselves** physically and in other ways when they use marijuana **ONCE A MONTH**, **regardless of whether or not pregnant**?

- ◯ No risk (1)
- ◯ Slight risk (7)
- ◯ Moderate risk (8)
- ◯ Great risk (9)
- ◯ I do not know (5)
- ◯ I do not wish to answer this question (6)

Page Break

Q38

What is your current opinion: How much do women risk **harming themselves** physically and in other ways when they use marijuana **ONCE OR TWICE A WEEK**, **regardless of whether or not pregnant**?

- ○ No risk (1)
- ○ Slight risk (7)
- ○ Moderate risk (8)
- ○ Great risk (9)
- ○ I do not know (5)
- ○ I do not wish to answer this question (6)

Page Break

Q39 **What is your current opinion:** How much do **pregnant women** risk **harming themselves** physically and in other ways when they use marijuana **ONCE A MONTH**?

- No risk (1)
- Slight risk (7)
- Moderate risk (8)
- Great risk (9)
- I do not know (10)
- I do not wish to answer this question (11)

Page Break

Q40 **What is your current opinion:** How much do **pregnant women** risk **harming themselves** physically and in other ways when they use marijuana **ONCE OR TWICE A WEEK**?

- No risk (1)
- Slight risk (7)
- Moderate risk (8)
- Great risk (9)
- I do not know (10)
- I do not wish to answer this question (11)

Page Break

Q41 **What is your current opinion:** Can marijuana use **ONCE A MONTH** during pregnancy **harm the baby**?

- ○ No risk (1)
- ○ Slight risk (8)
- ○ Moderate risk (9)
- ○ Great risk (10)
- ○ I do not know (11)
- ○ I do not wish to answer this question (12)

Page Break

Q42 **What is your current opinion:** Can marijuana use **ONCE OR TWICE A WEEK** during pregnancy **harm the baby**?

- ○ No risk (1)
- ○ Slight risk (8)
- ○ Moderate risk (9)
- ○ Great risk (10)
- ○ I do not know (11)
- ○ I do not wish to answer this question (12)

Page Break

Q43 **What is your current opinion:** Can marijuana use while breastfeeding harm the infant?

- ○ No risk (1)
- ○ Slight risk (8)
- ○ Moderate risk (9)
- ○ Great risk (10)
- ○ I do not know (11)
- ○ I do not wish to answer this question (12)

End of Block: Risk Perception

Start of Block: Delivery

Q44
The following questions are about your most recent pregnancy.

When did you give birth?

- ○ I gave birth this week (1)
- ○ I gave birth 1 week ago (2)
- ○ I gave birth 2 weeks ago (3)
- ○ I gave birth 3 weeks ago (4)
- ○ I gave birth 4 weeks ago (5)
- ○ Other. Please specify (9) ____

Page Break

Q45
Please recall that you were asked to record the following:

Gestational age is measured from the first day of your last menstrual period to determine the age (in weeks) of your baby.
At what **gestational age** did you give birth?

- ○ Less than 28 weeks (1)
- ○ 28 weeks (11)
- ○ 29 weeks (12)
- ○ 30 weeks (13)
- ○ 31 weeks (14)
- ○ 32 weeks (15)
- ○ 33 weeks (16)
- ○ 34 weeks (17)
- ○ 35 weeks (18)
- ○ 36 weeks (2)
- ○ 37 weeks (3)
- ○ 38 weeks (4)
- ○ 39 weeks (5)
- ○ 40 weeks (6)
- ○ 41 weeks (7)
- ○ 42 weeks or more (8)
- ○ I do not know (9)
- ○ I do not wish to answer this question (19)

Page Break

Q46 Please recall that you were asked to record the following:

By the end of your most recent pregnancy, how much did **YOU** weigh?

- ◯ Weight in pounds: (1) ____
- ◯ Weight in Kilograms: (2) ____
- ◯ I do not know (3)
- ◯ I do not wish to answer this question (4)

Q47 Was an **infection(s)** present during you most recent pregnancy?

- ▢ Yes, Chlamydia was present (1)
- ▢ Yes, Gonorrhea was present (3)
- ▢ Yes, Hepatitis B was present (4)
- ▢ Yes, Hepatitis C was present (5)
- ▢ Yes, Syphilis was present (6)
- ▢ Other. Please specify: (7) ____
- ▢ No infection was present (9)
- ▢ I do not know (10)
- ▢ I do not wish to answer this question (11)

Q48 Did you experience any **complications** during your most recent **pregnancy**?

- Yes. Please specify (1) ____
- No (2)
- I do not know (3)
- I do not wish to answer this question (4)

Display This Question:

If Did you experience any complications during your most recent pregnancy? = Yes. Please specify

Q49 Were **YOU** admitted to the **intensive care unit (ICU)** as a result of **complications** during your most recent **pregnancy**?

- Yes (1)
- No (2)
- I do not know (3)
- I do not wish to answer this question (4)

Display This Question:

If Were YOU admitted to the intensive care unit (ICU) as a result of complications during your most... = Yes

Q50 How many **days** did you stay in the **ICU** as a result of **complications** during your most recent **pregnancy**?

- Less than 1 day (1)
- Length of your ICU stay in DAYS: (2) ____
- I do not know (3)
- I do not wish to answer this question (4)

Page Break

Q51 Did you experience any **complications** at the time of **delivery**?

- ◯ Yes. Please specify (1) ____
- ◯ No (2)
- ◯ I do not know (3)
- ◯ I do not wish to answer this question (4)

Display This Question:

If Did you experience any complications at the time of delivery? = Yes. Please specify

Q52 Were **YOU** admitted to the **intensive care unit (ICU)** as a result of **complications** at the time of **delivery**?

- ◯ Yes (1)
- ◯ No (2)
- ◯ I do not know (3)
- ◯ I do not wish to answer this question (4)

Display This Question:

If Were YOU admitted to the intensive care unit (ICU) as a result of complications at the time of de... = Yes

Q53 How many **days** did you stay in the intensive care unit (ICU) as a result of complications at the time of delivery?

- ○ Less than 1 day (1)
- ○ Length of your ICU stay in DAYS: (2) ____
- ○ I do not know (3)
- ○ I do not wish to answer this question (4)

Page Break

Q54 Where did you give birth?

- In a hospital or birth center (1)
- At home (2)
- Other. Please specify (3) ____
- I do not wish to answer this question (4)

Q55 How long were you in labor?

- Less than 3 hours (1)
- 3-11 hours (2)
- 12-19 hours (3)
- Greater than 20 hours (4)
- I do not know (5)
- I do not wish to answer this question (6)

Page Break

Q56 I had a .....

◯ Vaginal delivery (1)

◯ Cesarean section (C-section), a surgical procedure in which incisions are made through the abdomen and uterus to deliver a baby. (2)

◯ Other. Please specify: (5) ____

◯ I do not wish to answer this question (4)

Q57
Please recall that you were asked to record the following:

Labor induction is the stimulation of uterine contractions during pregnancy before labor begins on its own.

Was your **labor induced**?

◯ Yes (1)

◯ No (2)

◯ I do not know (3)

◯ I do not wish to answer this question (4)

Display This Question:

If I had a ..... = Cesarean section (C-section), a surgical procedure in which incisions are made through the abdomen and uterus to deliver a baby.

Q58 My Cesarean section (C-section) was ....

- ○ Planned (1)
- ○ Emergency (2)
- ○ Other. Please specify: (3) ____
- ○ I do not know (4)
- ○ I do not wish to answer this question (5)

Display This Question:

If Where did you give birth? = In a hospital or birth center

Q59 How many **days** did **YOU stay in the hospital** after you delivered your baby?

- ○ Less than 1 day (2)
- ○ Length of your hospital stay in DAYS: (3) ____
- ○ I do not know (4)
- ○ I do not wish to answer this question (5)

Page Break

Q61 **How many** babies did you deliver?

- ◯ I gave birth to 1 new baby (singleton) (1)
- ◯ I gave birth to 2 new babies (twins) (2)
- ◯ I gave birth to 3 or more new babies (3)
- ◯ I do not know (4)
- ◯ I do not wish to answer this question (5)

Display This Question:

If How many babies did you deliver? = I gave birth to 1 new baby (singleton)

Q60 Has your delivery resulted in a **live** birth?

- ◯ Yes (1)
- ◯ No (2)

Page Break

Display This Question:

If How many babies did you deliver? = I gave birth to 1 new baby (singleton)

Q62 What is the **sex** of your new baby?

○ Male (1)

○ Female (2)

Page Break

Display This Question:

If How many babies did you deliver? = I gave birth to 1 new baby (singleton)

Q63 Please recall that you were asked to record the following:

When your baby was born, what was his/her **birthweight**?

- ◯ Birthweight in pounds (e.g., 7 lbs. 6 oz.): (1) ____
- ◯ Birthweight in grams (e.g., 3345 g): (2) ____
- ◯ I do not know (3)
- ◯ I do not wish to answer this question (4)

Page Break

Display This Question:

If How many babies did you deliver? = I gave birth to 1 new baby (singleton)

Q64
The **neonatal intensive care unit**, or **NICU**, is a nursery in a hospital that provides care to sick or premature newborns following birth.

Was your new baby **admitted to the NICU** following birth?

- ◯ Yes (1)
- ◯ No (2)
- ◯ I do not know (3)
- ◯ I do not wish to answer this question (4)

Display This Question:

If The neonatal intensive care unit, or NICU, is a nursery in a hospital that provides care to sick... = Yes

Q65 How many **days** did your baby stay in the **neonatal intensive care unit (NICU)**?

- ◯ Less than 1 day (4)
- ◯ Length of newborn's hospital stay in DAYS: (1) ____
- ◯ I do not know (2)
- ◯ I do not wish to answer this question (3)

Page Break

Display This Question:

If How many babies did you deliver? = I gave birth to 1 new baby (singleton)

Q66
Please recall that you were asked to record the following:

An **Apgar score** provides a standardized assessment for infants after delivery. It measures five components: the infant's 1) color, 2) heart rate, 3) reflexes, 4) muscle tone, and 5) respiration.

The **Apgar score** is measured **1 minute** after birth and ranges from 0 to 10.

What was your infant's **1-MINUTE Apgar score**?

▼ 0 (1) ... I do not wish to answer this question (13)

Page Break

Display This Question:

If How many babies did you deliver? = I gave birth to 1 new baby (singleton)

Q67
Please recall that you were asked to record the following:

An **Apgar score** provides a standardized assessment for infants after delivery. It measures five components: the infant's 1) color, 2) heart rate, 3) reflexes, 4) muscle tone, and 5) respiration.

The **Apgar score** is also measured **5 minutes** after birth and ranges from 0 to 10.

What was your infant's **5-MINUTE Apgar score**?

▼ 0 (1) ... I do not wish to answer this question (13)

Page Break

Display This Question:

If How many babies did you deliver? = I gave birth to 2 new babies (twins)

Or How many babies did you deliver? = I gave birth to 3 or more new babies

Q128 Has your delivery resulted in a live birth of baby #1?

- ○ Yes (1)
- ○ No (2)

Display This Question:

If How many babies did you deliver? = I gave birth to 2 new babies (twins)

Or How many babies did you deliver? = I gave birth to 3 or more new babies

Q68 What is the **sex** of baby #1?

- ○ Male (1)
- ○ Female (2)

Page Break

Display This Question:

If How many babies did you deliver? = I gave birth to 2 new babies (twins)

Or How many babies did you deliver? = I gave birth to 3 or more new babies

Q69 Please recall that you were asked to record the following:

When baby #1 was born, what was his/her **birthweight**?

○ Birthweight in pounds (e.g., 7 lbs. 6 oz.): (1) ____

○ Birthweight in grams (e.g., 3345 g): (2) ____

○ I do not know (3)

○ I do not wish to answer this question (4)

Page Break

Display This Question:

If How many babies did you deliver? = I gave birth to 2 new babies (twins)

Or How many babies did you deliver? = I gave birth to 3 or more new babies

Q70
The **neonatal intensive care unit**, or **NICU**, is a nursery in a hospital that provides care to sick or premature newborns following birth.

Was baby #1 **admitted to the NICU** following birth?

◯ Yes (1)

◯ No (2)

◯ I do not know (3)

◯ I do not wish to answer this question (4)

Display This Question:

If The neonatal intensive care unit, or NICU, is a nursery in a hospital that provides care to sick... = Yes

Q71 How many **days** did baby #1 stay in the **neonatal intensive care unit (NICU)**?

◯ Less than 1 day (4)

◯ Length of newborn's hospital stay in DAYS: (1) ____

◯ I do not know (2)

◯ I do not wish to answer this question (3)

Page Break

Display This Question:

If How many babies did you deliver? = I gave birth to 2 new babies (twins)

Or How many babies did you deliver? = I gave birth to 3 or more new babies

Q72
Please recall that you were asked to record the following:

An **Apgar score** provides a standardized assessment for infants after delivery. It measures five components: the infant's 1) color, 2) heart rate, 3) reflexes, 4) muscle tone, and 5) respiration.

The **Apgar score** is measured **1 minute** after birth and ranges from 0 to 10.

What was baby #1's **1-MINUTE Apgar score**?

▼ 0 (1) ... I do not wish to answer this question (13)

Page Break

Display This Question:

If How many babies did you deliver? = I gave birth to 2 new babies (twins)

Or How many babies did you deliver? = I gave birth to 3 or more new babies

Q73
Please recall that you were asked to record the following:

An **Apgar score** provides a standardized assessment for infants after delivery. It measures five components: the infant's 1) color, 2) heart rate, 3) reflexes, 4) muscle tone, and 5) respiration.

The **Apgar score** is also measured **5 minutes** after birth and ranges from 0 to 10.

What was baby #1's **5-MINUTE Apgar score**?

▼ 0 (1) ... I do not wish to answer this question (13)

Page Break

Display This Question:

If How many babies did you deliver? = I gave birth to 2 new babies (twins)

Or How many babies did you deliver? = I gave birth to 3 or more new babies

Q129 Has your delivery resulted in a live birth of baby #2?

- ◯ Yes (1)
- ◯ No (2)

Display This Question:

If How many babies did you deliver? = I gave birth to 2 new babies (twins)

Or How many babies did you deliver? = I gave birth to 3 or more new babies

Q74 What is the **sex** of baby #2?

- ◯ Male (1)
- ◯ Female (2)

Page Break

Display This Question:

If How many babies did you deliver? = I gave birth to 2 new babies (twins)

Or How many babies did you deliver? = I gave birth to 3 or more new babies

Q75 Please recall that you were asked to record the following:

When baby #2 was born, what was his/her **birthweight**?

◯ Birthweight in pounds (e.g., 7 lbs. 6 oz.): (1) ____

◯ Birthweight in grams (e.g., 3345 g): (2) ____

◯ I do not know (3)

◯ I do not wish to answer this question (4)

Page Break

Display This Question:

If How many babies did you deliver? = I gave birth to 2 new babies (twins)

Or How many babies did you deliver? = I gave birth to 3 or more new babies

Q76
The **neonatal intensive care unit**, or **NICU**, is a nursery in a hospital that provides care to sick or premature newborns following birth.

Was baby #2 **admitted to the NICU** following birth?

○ Yes (1)

○ No (2)

○ I do not know (3)

○ I do not wish to answer this question (4)

Display This Question:

If The neonatal intensive care unit, or NICU, is a nursery in a hospital that provides care to sick... = Yes

Q77 How many **days** did baby #2 stay in the **neonatal intensive care unit (NICU)**?

○ Less than 1 day (4)

○ Length of newborn's hospital stay in DAYS: (1) ____

○ I do not know (2)

○ I do not wish to answer this question (3)

Page Break

Display This Question:

If How many babies did you deliver? = I gave birth to 2 new babies (twins)

Or How many babies did you deliver? = I gave birth to 3 or more new babies

Q78
Please recall that you were asked to record the following:

An **Apgar score** provides a standardized assessment for infants after delivery. It measures five components: the infant's 1) color, 2) heart rate, 3) reflexes, 4) muscle tone, and 5) respiration.

The **Apgar score** is measured **1 minute** after birth and ranges from 0 to 10.

What was baby #2's **1-MINUTE Apgar score**?

▼ 0 (1) ... I do not wish to answer this question (13)

Page Break

Display This Question:

If How many babies did you deliver? = I gave birth to 2 new babies (twins)

Or How many babies did you deliver? = I gave birth to 3 or more new babies

Q79
Please recall that you were asked to record the following:

An **Apgar score** provides a standardized assessment for infants after delivery. It measures five components: the infant's 1) color, 2) heart rate, 3) reflexes, 4) muscle tone, and 5) respiration.

The **Apgar score** is also measured **5 minutes** after birth and ranges from 0 to 10.

What was baby #2's **5-MINUTE Apgar score**?

▼ 0 (1) ... I do not wish to answer this question (13)

Page Break

Display This Question:

If How many babies did you deliver? = I gave birth to 3 or more new babies

Q130 Has your delivery resulted in a live birth of baby #3?

- Yes (1)
- No (2)

Display This Question:

If How many babies did you deliver? = I gave birth to 3 or more new babies

Q80 What is the **sex** of baby #3?

- Male (1)
- Female (2)

Page Break

Display This Question:

If How many babies did you deliver? = I gave birth to 3 or more new babies

Q81 Please recall that you were asked to record the following:

When baby #3 was born, what was his/her **birthweight**?

○ Birthweight in pounds (e.g., 7 lbs. 6 oz.): (1) ____

○ Birthweight in grams (e.g., 3345 g): (2) ____

○ I do not know (3)

○ I do not wish to answer this question (4)

Page Break

Display This Question:

If How many babies did you deliver? = I gave birth to 3 or more new babies

Q82
The **neonatal intensive care unit**, or **NICU**, is a nursery in a hospital that provides care to sick or premature newborns following birth.

Was baby #3 **admitted to the NICU** following birth?

- ◯ Yes (1)
- ◯ No (2)
- ◯ I do not know (3)
- ◯ I do not wish to answer this question (4)

Display This Question:

If The neonatal intensive care unit, or NICU, is a nursery in a hosptal that provides care to sick o... = Yes

Q83 How many **days** did baby #3 stay in the **neonatal intensive care unit (NICU)**?

- ◯ Less than 1 day (4)
- ◯ Length of newborn's hospital stay in DAYS: (1) ____
- ◯ I do not know (2)
- ◯ I do not wish to answer this question (3)

Page Break

Display This Question:

If How many babies did you deliver? = I gave birth to 3 or more new babies

Q84
Please recall that you were asked to record the following:

An **Apgar score** provides a standardized assessment for infants after delivery. It measures five components: the infant's 1) color, 2) heart rate, 3) reflexes, 4) muscle tone, and 5) respiration.

The **Apgar score** is measured **1 minute** after birth and ranges from 0 to 10.

What was baby #3's **1-MINUTE Apgar score**?

▼ 0 (1) ... I do not wish to answer this question (13)

Page Break

Display This Question:

If How many babies did you deliver? = I gave birth to 3 or more new babies

Q85
Please recall that you were asked to record the following:

An **Apgar score** provides a standardized assessment for infants after delivery. It measures five components: the infant's 1) color, 2) heart rate, 3) reflexes, 4) muscle tone, and 5) respiration.

The **Apgar score** is also measured **5 minutes** after birth and ranges from 0 to 10.

What was baby #3's **5-MINUTE Apgar score**?

▼ 0 (1) ... I do not wish to answer this question (13)

Page Break

Display This Question:

If Has your delivery resulted in a live birth? = Yes

Q86 Is your baby living with you now?

- Yes (1)
- No (2)
- I do not wish to answer this question (3)

Display This Question:

If Is your baby living with you now? = Yes

Q87 Did you ever breastfeed or pump breast milk to feed your new baby, even for a short period of time?

- Yes (1)
- No (2)
- I do not wish to answer this question (3)

Display This Question:

If Did you ever breastfeed or pump breast milk to feed your new baby, even for a short period of time? = Yes

Q88 Are you currently breastfeeding or feeding pumped milk to your new baby?

- Yes (1)
- No (2)
- I do not wish to answer this question (4)

End of Block: Delivery
